# Comparative DNA Damage and Repair in Echinoderm Coelomocytes Exposed to Genotoxicants

**DOI:** 10.1371/journal.pone.0107815

**Published:** 2014-09-17

**Authors:** Ameena H. El-Bibany, Andrea G. Bodnar, Helena C. Reinardy

**Affiliations:** Molecular Discovery Laboratory, Bermuda Institute of Ocean Sciences, St. George's, Bermuda; University of Gothenburg, Sweden

## Abstract

The capacity to withstand and repair DNA damage differs among species and plays a role in determining an organism's resistance to genotoxicity, life history, and susceptibility to disease. Environmental stressors that affect organisms at the genetic level are of particular concern in ecotoxicology due to the potential for chronic effects and trans-generational impacts on populations. Echinoderms are valuable organisms to study the relationship between DNA repair and resistance to genotoxic stress due to their history and use as ecotoxicological models, little evidence of senescence, and few reported cases of neoplasia. Coelomocytes (immune cells) have been proposed to serve as sensitive bioindicators of environmental stress and are often used to assess genotoxicity; however, little is known about how coelomocytes from different echinoderm species respond to genotoxic stress. In this study, DNA damage was assessed (by Fast Micromethod) in coelomocytes of four echinoderm species (sea urchins *Lytechinus variegatus*, *Echinometra lucunter lucunter*, and *Tripneustes ventricosus*, and a sea cucumber *Isostichopus badionotus*) after acute exposure to H_2_O_2_ (0–100 mM) and UV-C (0–9999 J/m^2^), and DNA repair was analyzed over a 24-hour period of recovery. Results show that coelomocytes from all four echinoderm species have the capacity to repair both UV-C and H_2_O_2_-induced DNA damage; however, there were differences in repair capacity between species. At 24 hours following exposure to the highest concentration of H_2_O_2_ (100 mM) and highest dose of UV-C (9999 J/m^2^) cell viability remained high (>94.6±1.2%) but DNA repair ranged from 18.2±9.2% to 70.8±16.0% for H_2_O_2_ and 8.4±3.2% to 79.8±9.0% for UV-C exposure. Species-specific differences in genotoxic susceptibility and capacity for DNA repair are important to consider when evaluating ecogenotoxicological model organisms and assessing overall impacts of genotoxicants in the environment.

## Introduction

There has been much interest to integrate assessment of genetic effects into environmental studies to broaden the understanding of ecotoxicological impacts on organisms and populations [1]–[4]. Maintenance of DNA integrity is essential for proper cellular and organismal function, and the capacity to withstand genotoxic challenge is important to avoid long-term genetic instability and population vulnerability [5]. Unrepaired DNA damage can lead to mutations, cellular senescence, apoptosis, progression of cancer [6], and the process of aging [7]. Of particular concern in ecotoxicology is the potential for chronic effects and trans-generational impacts on populations by transfer of damaged DNA to offspring [8]. To minimize the harmful consequences of DNA damage, organisms are equipped with a variety of cellular defense and DNA repair mechanisms.

DNA is constantly damaged by both endogenous and exogenous sources, and genotoxicity can be considered as an imbalance between DNA damage and DNA repair mechanisms. Two major model genotoxicants are ultraviolet (UV) radiation and hydrogen peroxide (H_2_O_2_), which each induce different forms of DNA lesions. UV-C (<280 nm) is absorbed by the ozone in the earth's atmosphere and UV-B is the main component of UV radiation of environmental concern [9]; however, both UV-B and UV-C induce formation of cyclobutane-pyrimidine dimers (CPDs) and 6-4 photoproducts (6-4PPs) [10], in addition to DNA strand breaks [11]. UV-C induces high levels of DNA damage [12] and is commonly used as a model genotoxicant to investigate biological effects of UV irradiation [13], [14]. H_2_O_2_ is produced as a byproduct of metabolic processes and cellular defense mechanisms [15], and is an important reactive oxygen species (ROS) involved in exogenously-induced oxidative DNA damage [16]. Antioxidant activity can restrict oxidative DNA lesions to several hundred per day, but excess ROS or a deficiency in antioxidants can lead to increased base oxidation and DNA strand breaks [17]. UV- and H_2_O_2_-induced DNA damage are primarily repaired by nucleotide excision repair (NER) and base excision repair (BER), respectively [18], [14]. Investigation of DNA damage and repair after exposure to these two genotoxicants can inform on susceptibility to both oxidative damage and UV-induced DNA lesions, in addition to the capacity for both BER and NER.

Marine invertebrates have been extensively studied as bioindicators of environmental stress [19], and the sea urchin embryo test has served as a sensitive indicator of pollutant genotoxicology, embryo-toxicology, and teratogenicity [20]–[23]. Activation of DNA damage checkpoints, DNA repair, and apoptosis in sea urchin embryos have been demonstrated in response to genotoxicants such as methyl methanesulfonate, bleomycin, and exposure to ultraviolet radiation [24]–[26]. Despite the fact that sea urchin embryos are frequently used in toxicity testing, little is known of the effects of genotoxicants on the cells of adult sea urchins. Information about the cellular response of adult sea urchins to environmental stress is valuable for ecotoxicological studies and would increase understanding of the life history traits of these animals. Life history studies show that different species of sea urchins exhibit a very large range of reported lifespans (from approximately 3 to more than 100 years) [27]–[30], there is little evidence of senescence [31], and few reported cases of neoplasia [32], [33]. Investigating DNA damage and DNA repair in cells of different sea urchin species would provide valuable information on selection of appropriate bioindicator species, allow assessments of environmental stress on different species, and shed light on mechanisms underlying life history traits of these animals.

The open circulatory system of echinoderms is comprised of coelomic fluid containing different cells types, collectively termed ‘coelomocytes’. Coelomocytes fall into one of three categories: phagocytes, spherule cells (red and colorless), and vibratile cells, with further sub-categories within each cell type [34]. Coelomocytes play an integral role in immune cell functions such as fighting microbial infections and wound healing [34]. Damage to coelomocytes can compromise these essential functions, directly affecting the health of organisms and stability of populations. Coelomocytes (or circulating cells) from a variety of terrestrial and aquatic organisms (e.g. earthworms, bivalves, fish) have been useful bioindicators of environmental stress and are frequently used to assess genotoxicity [35]–[40]. Changes in the number and/or composition of coelomocytes have been reported in sea urchins from contaminated environments and those exposed to elevated *p*CO2 or increased temperature, suggesting that sea urchin coelomocytes may also serve as sensitive indicators of environmental stress [9], [41]–[44]. However, another study showed that DNA from coelomocytes of the sea urchin *Lytechinus variegatus* is relatively resistant to genotoxicants [45]. Understanding susceptibility to DNA damage and DNA repair capacity of coelomocytes from different echinoderm species would be useful in assessing the value of coelomocytes as bioindicator cells and understanding the overall impacts of genotoxicants on these organisms. Persistent genotoxic damage is dependent on the balance between repair and replacement of damaged cells. Studies on echinoderms indicate a low level of cell turnover in the coelomocyte population (<1.5% BrdU incorporation in 3 hours [46] or 16 hours [47] in star fish) and low levels of apoptosis following acute exposures to UV-B [9], UV-C, hydrogen peroxide, methylmethane sulfonate and benzo [a]pyrene [45]; however, the DNA repair capacity of coelomocytes from different echinoderm species has not been investigated.

The objectives of this study are to assess the capacity to which cells from different echinoderm species are able to repair different types of DNA damage after exposure to two model genotoxicants, UV-C and H_2_O_2_. The specific aims are to comparatively evaluate the DNA damage and DNA repair capabilities in coelomocytes of four echinoderm species (sea urchins *L. variegatus*, *Echinometra lucunter lucunter*, *Tripneustes ventricosus*, and sea cucumber *Isostichopus badionotus*). We hypothesize that echinoderm coelomocytes will be able to repair some level of DNA damage, and the extent of genotoxicity sensitivity and DNA repair capacity will differ among species.

## Materials and Methods

### Animal collection and maintenance

All animals were collected and maintained in strict accordance with the Collecting and Experimental Ethics Policy (CEEP) of the Bermuda Institute of Ocean Sciences. All experiments complied with the ethical policy of the CEEP committee and did not require specific approval. All experiments were carried out on coelomocytes extracted from animals with minimal impact, except for a single small *E. l. lucunter* which was sacrificed in order to collect sufficient coelomic fluid for the experiment, and all efforts were made to minimize suffering. Except as mentioned above, all animals showed no adverse behavioral effects of the coelomocyte sampling procedure, all animals survived the procedure, and all animals were returned to their collection location.

Collection of animals complied with the collection policy of CEEP, no species were endangered, and no animals were collected from protected locations. Collection numbers of *L. variegatus*, *I. badionotus*, and *T. ventricosus* were within the CEEP collection limits and no specific collection permission was required. Collection of *E. l. lucunter* was carried out under a Department of Environmental Protection special permit (permit no, 131002, Bermuda Government), approved by the Director of Environmental Protection. All species were collected from the shallow sub-littoral zone (less than 2 m depth at low tide), September–October, 2013, in Bermuda. *L. variegatus* and *I. badionotus* were collected from Harrington Sound (32°19.4′N, 64°43.6′W), *T. ventricosus* were collected from Fort St. Catherine beach (32°23.3′N, 64°40.3′W), and *E. l. lucunter* were collected from Castle Harbor (32°21.2′N, 64°39.8′W) and Gravelly Bay (32°19.1′N, 64°42.8′W). Animal husbandry and maintenance complied with CEEP policy. Sea urchins were maintained in flow-through aquaria with ambient temperature and light, and were left to acclimate for a minimum of 1 week after collection. *I. badionotus* were maintained in an outdoor flow-through aquarium with a layer of sediment on the bottom, and were left to acclimate for 1 week. Sea urchins were fed weekly with fresh sea grass, and sediment was replenished fortnightly in the *I. badionotus* aquarium.

### Coelomocyte collection and treatment

Unless otherwise specified, all chemicals were sourced from Sigma-Aldrich (Sigma-Aldrich Co., St. Louis, MO, USA). Sea urchin test diameter was measured with calipers, and 2–6 ml coelomic fluid was extracted by syringe with an 18-guage needle inserted through the peristomial membrane surrounding the Aristotle's lantern. Sea cucumber size was estimated by weight, width, and length measurements, and 6–10 ml coelomic fluid was extracted by syringe with a 21-guage needle inserted laterally in the mid-body region. The experiments were designed to include a single coelomocyte collection per animal, division of the coelomic fluid for UV-C or H_2_O_2_ treatment, and proceeding concurrently with exposure and recovery period of both sets of treatment samples. Cell concentration, cell viability, and differential cell counts (red and other coelomocytes) were calculated after 1∶1 dilution with trypan blue [0.8% trypan blue in calcium- magnesium-free seawater (CMFSW: 460 mM NaCl, 10 mM KCL, 7 mM Na_2_SO_4_, 2.4 mM NaHCO_3_, pH 7.4) containing 30 mM EDTA] using a haemocytometer (Neubauer Bright Line haemocytometer). The volume for 50,000 cells per assay reaction (in triplicate or quadruplicate) was estimated and aliquoted into microcentrifuge tubes for each exposure. From the species selected for this study, cell aggregation was not a considerable factor in the experimental set-up. Coelomocytes from *L. variegatus*, *T. ventricosus*, and *I. badionotus* did not exhibit a strong agglutination reaction and could easily be dissociated to single cell suspensions by gently pipetting or vortexing. *E. l. lucunter* coelomocytes did exhibit some aggregation but clumps of cells were avoided when sample aliquots were taken. Differential cell counts and cell viability were estimated on all control and highest-exposed (9999 J/m^2^ and 100 mM for UV-C and H_2_O_2_, respectively) samples after 24 hours recovery.

For the UV-C (254 nm) treatment, coelomocyte samples (25–132 µl volume) were irradiated (0, 250, 1000, 3000, or 9999 J/m^2^) in 0.5 ml open microcentrifuge tubes in a Stratalinker UV Crosslinker 1800 (Stratagene, La Jolla, CA, USA). The recovery period was timed to begin immediately after dose delivery, and samples were left to recover for 0, 1, 3, 6, and 24 hours in the dark at room temperature. At each recovery timepoint, samples were placed on ice to halt DNA repair and processed for the Fast Micromethod assay.

For the H_2_O_2_ treatment, coelomocyte samples were exposed in 1.5 ml microcentrifuge tubes. H_2_O_2_ stock dilutions were prepared in CMFSW and added to coelomocyte samples to give the following final concentrations: 0, 0.1, 1, 10, or 100 mM H_2_O_2_. Samples were left in the dark for 10 min followed by 5 min centrifugation (8000 *g*) at room temperature. H_2_O_2_ exposure was halted by removal of supernatant after centrifugation, and cells were re-suspended in cell-free coelomic fluid (CFCF, prepared by collection of supernatant after centrifugation, 13000 *g* for 5 min, of coelomic fluid to remove cells) and the recovery period was started. At each recovery timepoint, samples were placed on ice to halt DNA repair, and processed for the Fast Micromethod assay.

### Fast Micromethod for estimation of DNA damage

The method for fluorescent detection of alkaline DNA unwinding was carried out as described by Schröder et al. [48], with minor modifications. In brief, samples were assayed after respective periods of recovery and coelomocyte sample volume was adjusted with CFCF to make up to 50,000 cells per reaction. Samples were assayed in triplicate or quadruplicate by loading 20 µl sample to each replicate well on a black-walled 96-well microplate (USA Scientific, Inc., Ocala, FL, USA), and 20 µl of suitable blank (CMFSW or CFCF) were added to control wells. In some instances for *L. variegatus*, fewer cells were used per reaction when the cell concentration in coelomic fluid was low. Cells were lysed by adding 20 µl of lysing solution (9.0 M urea, 0.1% SDS, 0.2 M EDTA) containing 1∶49 PicoGreen (Life Technologies, Grand Island, NY, USA, P7581), and left in the dark on ice for 40 min. DNA unwinding solution (20 mM EDTA, 1 M NaOH) was added (200 µl) to initiate alkaline unwinding (pH 12.4±0.02), fluorescence was detected (kinetic mode, excitation 480 nm, emission 520 nm, SpectraMax M2 Microplate Reader, Molecular Devices, CA, USA), and relative fluorescent units (RFU) was recorded every 5 min for a 30-min period. DNA unwinding was carried out at room temperature.

DNA damage was calculated according to the strand scission factor (SSF) equation [48]: SSF = log (% dsDNA_sample_/% dsDNA_control_)×(−1), where dsDNA_sample_ are the treated samples and dsDNA_control_ are the unexposed samples, and percentages are calculated from RFU after 20-min unwinding compared with initial (0 min unwinding) RFU, after subtracting respective blank RFU (CMFSW or CFCF). Due to high background fluorescence in CFCF from *I. badionotus*, RFU for that species were blanked with CMFSW RFU, but other species' RFU were blanked with individual CFCF RFU.

### Analyses

Both treatments (UV-C and H_2_O_2_) were conducted concurrently on a single coelomocyte sample per animal, and different animals (*T. ventricosus* n = 5, *L. variegatus* n = 12, *E. l. lucunter* n = 8, and *I. badionotus* n = 8) were considered biological replicates in all analyses. DNA damage estimation by Fast Micromethod included technical replicates (n = 3–4) of each sample to give an overall SSF per sample for each animal, and all biological replicates were combined for analyses of coelomocyte parameters, initial dose/concentration response, and DNA damage profiles over the 24-hour period of recovery.

Statistical analyses were performed in Statgraphics Centurion XVI.I (StatPoint Technologies, Inc., VA, USA). Intraspecific effects of size on DNA damage (SSF) during the 24-hour recovery period was tested by general linear model (GLM) with test diameter (average length for *I. badionotus*), dose/concentration, and recovery time as quantitative variables. To investigate intraspecific effects of concentration/dose and time, all individuals within a species were combined and DNA damage (SSF) was tested by GLM with concentration/dose and time as quantitative independent variables; dose/concentration differences from controls after 24 hours recovery were tested by one-way ANOVA or Kruskal-Wallis (for normally distributed or non-normally distributed data, respectively), with *post-hoc* Fisher's least significant difference (LSD) test at the 95% confidence level. Differences in DNA repair between species were tested by GLM, with species as a categorical factor, and concentration/dose and time as quantitative independent variables; species differences were established by *post-hoc* multiple range tests. Additionally, DNA repair was estimated as the percentage of DNA damage after 24 hours recovery compared with initial (0-hours recovery) DNA damage for each individual and for each exposure level, following the equation: % DNA repair = 100−((T_24_ SSF/T_0_ SSF)×100), where T_24_ SSF is SSF after 24 hours recovery, and T_0_ SSF is the initial (0-hours recovery) SSF; negative DNA repair values indicated no DNA repair and were set to zero. DNA repair (%) data was arcsine transformed to test for intraspecific differences in repair capacity (ANOVA, *post-hoc* multiple range tests). DNA repair capacity was categorized as follows: low (<25% DNA repair), moderate (25–50% DNA repair), high (50–75% DNA repair), or very high (>75% DNA repair).

## Results

No anti-coagulant was used for collection of coelomic fluid and there was minimal or no cell aggregation in coelomocytes from *L. variegatus*, *T. ventricosus*, and *I. badionotus*. A proportion of coelomocytes from *E. l. lucunter* aggregated within the first few minutes after collection, clumps were disaggregated by gently pipetting before analysis, and persistent clumps were avoided. Coelomocytes isolated from the different species were evaluated for cell concentration, proportion of white to red cells, and cell viability. *E. l. lucunter* and *I. badionotus* had significantly higher total coelomocyte concentrations compared with the other species (Kruskal-Wallis and multiple range test, p<0.05), and no red coelomocytes were observed in any sample from *I. badionotus* (Table 1). There was no significant cell death in any of the coelomocyte samples over the course of the study, with cell viability >94% 24 hours after exposure to UV-C or H_2_O_2_ (Table 1). A slight reduction in overall coelomocyte size was observed after 24 hours recovery from the highest levels of UV-C and H_2_O_2_.

**Table 1:** Number of individuals, size ranges, coelomocyte characterization, and cell viability after 24-hours recovery from highest levels exposures to UV-C and H_2_O_2_ of all echinoderms tested.

Species	n	Test diameter range (mm)	Pre-treatment				Viability 24 hours recovery after treatment (% of total cells)	
			Coelomocyte concentration (cells/µl)		Red coelomocytes (% of total cells)		UV-C (9999 J/m^2^)	H_2_O_2_ (100 mM)
*T. ventricosus*	5	101–115	1855±280	A	7.6±2.4	C	99.8±0.2	94.6±1.2
*L. variegatus*	12	47–85	1957±220	A	9.1±1.6	C	98.5±0.4	98.4±0.6
*E. l. lucunter*	8	27–71	4565±745	B	8.0±3.1	C	99.4±0.3	99.7±0.1
*I. badionotus*	8	87–258*	4386±839	B	0	D	99.2±0.3	98.1±0.5

*Length (mm, average of several measurements during sampling) was measured for *I. badionotus*. Different letters (A/B/C/D) denote significant interspecific difference in means (multiple range tests, p<0.05).

Data are mean ± s.e.m.

**Figure 1:**
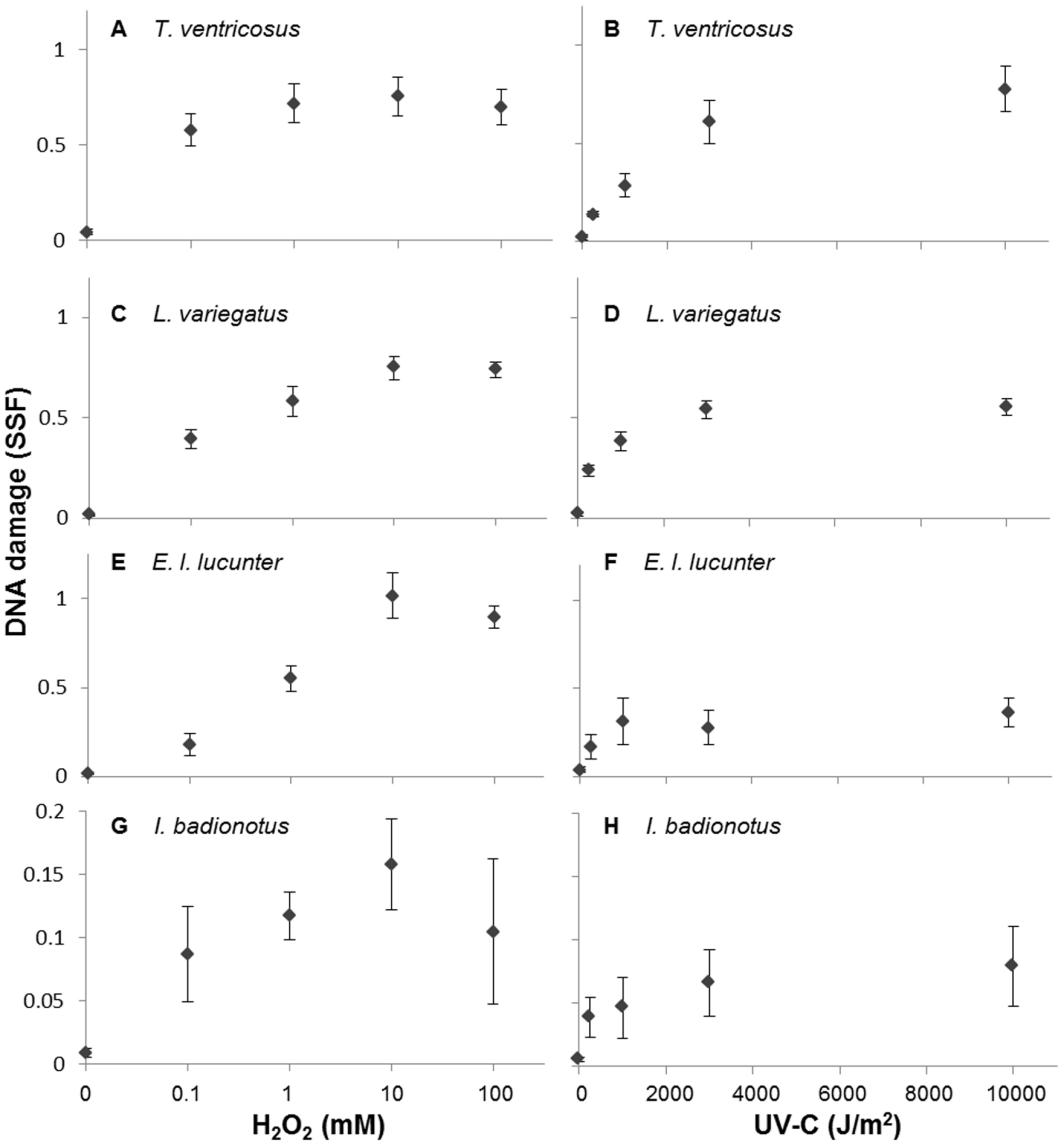
Dose/concentration response in echinoderm coelomocytes. Increase in DNA damage (strand scission factor, SSF, Fast Micromethod) with increasing concentration of H_2_O_2_ (**A**, **C**, **E**, and **G**) or dose of UV-C (**B**, **D**, **F**, and **H**) after acute exposure of coelomocytes from *T. ventricosus* (**A** and **B**, n = 5), *L. variegatus* (**C** and **D**, n = 11–12), *E. l. lucunter* (**E** and **F**, n = 6–7), and *I. badionotus* (**G** and **H**, n = 8). Data are means ± s.e.m.

Coelomocytes from all species showed an increase in DNA damage with increasing concentration or dose of genotoxicant (Figure 1). Patterns of dose responses indicated higher sensitivity in *T. ventricosus* and lower sensitivity in *E. l. lucunter* coelomocytes exposed to H_2_O_2_. *I. badionotus* had a lower magnitude of DNA damage after both genotoxicant treatments compared with the sea urchin species, and there was considerable inter-individual variation. The different sea urchin species responses to UV-C exposure were similar, with a slight indication of higher DNA damage at the highest doses in *T. ventricosus*.

Individuals of each species varied in size but there was no significant size effect over the 24-hour period of recovery after exposure to either H_2_O_2_ or UV-C in *T. ventricosus*, *L. variegatus*, *E. l. lucunter* (H_2_O_2_ only) or *I. badionutus* (GLM, p>0.05). There was a significant effect of size of DNA damage in *E. l. lucunter* after exposure to UV-C; however, the sample size was small and only 3 large individuals were collected therefore the biological significance is unknown and all individuals were grouped for further analyses.

**Figure 2:**
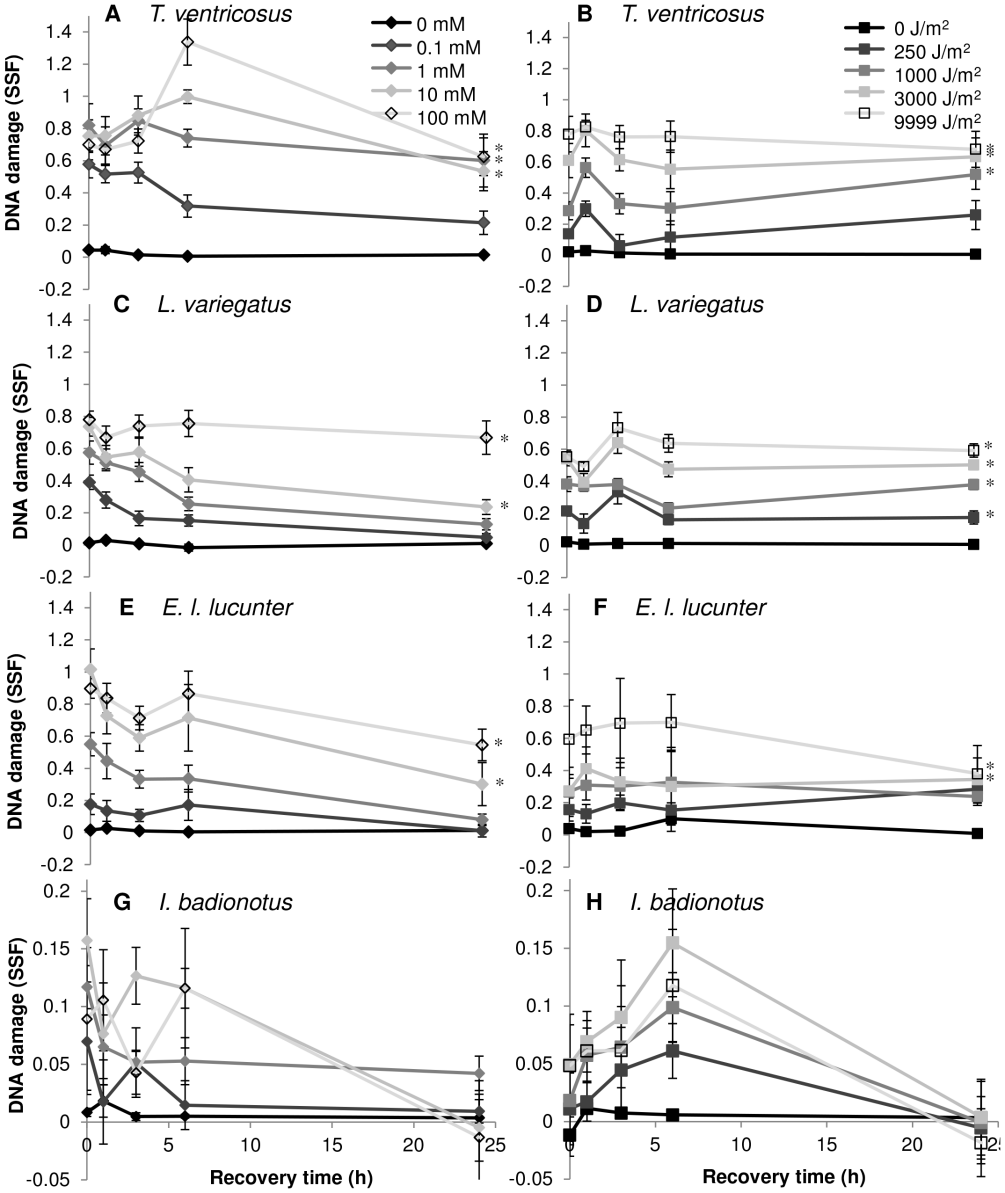
DNA repair in echinoderm coelomocytes. DNA repair [reduction in DNA damage (SSF)] over a 24-hour period of recovery after acute exposure to H_2_O_2_ (**A**, **C**, **E**, and **G**) or UV-C (**B**, **D**, **F**, and **H**) in coelomocytes from *T. ventricosus* (**A** and **B**, n = 5), *L. variegatus* (**C** and **D**, n = 12), *E. l. lucunter* (**E** and **F**, n = 8), and *I. badionotus* (**G** and **H**, n = 8). Data are means ± s.e.m. *Significantly higher than controls, indicating incomplete repair (within 24-hour timepoint, Fisher's LSD, p<0.05).

Each species had a different response in reduction in DNA damage over a 24-hour period of recovery after exposure to UV-C, however *L. variegatus* and *E. l. lucunter* were not different from each other after exposure to H_2_O_2_ (Figure 2, GLM p<0.05, *post-hoc* multiple range test). The temporal pattern of DNA damage over time was consistent among species, with clear DNA repair for most treatment levels for both exposures only evident after 6–24 hours recovery, and *I. badionotus* had greater inter-individual variation compared with the sea urchin species (Figure 2). None of the sea urchin species showed very high repair of DNA damage in the highest two exposures (10 and 100 mM H_2_O_2_, and 3000 and 9999 J/m^2^ UV-C) after 24 hours recovery, however *I. badionotus* showed high (>55%) or very high (>75%) repair of DNA damage at all exposure levels after 24 hours recovery (Figure 2, *post-hoc* Fisher's LSD, p<0.05, Table 2). *T. ventricosus* had highest DNA repair 24 hours after exposure to 0.1 mM H_2_O_2_ (59%) and 250 J/m^2^ UV-C (20%), compared with controls, but *L. variegatus* and *E. l. lucunter* had high (>65%) DNA repair up to 10 mM H_2_O_2_, and *E. l. lucunter* had moderate (38%) DNA repair at 3000 J/m^2^ UV-C.

**Table 2:** Percent DNA repair (DNA damage at 24 hours recovery compared with initial DNA damage†) in echinoderm coelomocytes after 24 hours recovery from acute exposure to H_2_O_2_ or UV-C.

		*T. ventricosus* (n = 5)	*L. variegatus* (n = 12)	*E. l. lucunter* (n = 7–8)	*I. badionotus* (n = 8)
**H_2_O_2_ (mM)**	0.1	58.6±12.2	81.8±5.4	59.6±17.6	57.8±17.0
	1	33.4±19.8	73.2±6.8	79.8±5.2	59.0±13.0
	10	32.6±8.2	65.0±7.2	71.2±7.8	83.4±12.2
	100	18.2±9.2	24.8±6.6*	41.8±8.4	70.8±16.0
**UV-C (J/m^2^)**	250	20.0±20.0	35.2±10.4	23.6±11.8	54.8±17.6
	1000	11.0±11.0	13.2±4.4	27.0±15.8	67.0±16.2
	3000	15.6±6.6	13.6±3.8	38.0±18.0	61.2±17.0
	9999	16.2±15.8	8.4±3.2	53.2±15.8	79.8±9.0

† % DNA repair = 100−((T_24_ SSF/T_0_ SSF)×100), where T_24_ SSF is strand scission factor (SSF) after 24 hours recovery and T_0_ SSF is the initial (0-hour) recovery SSF; negative % DNA repair values indicated no DNA repair and were set to zero.

*Significant reduction in DNA repair within a species (arcsine transformed, ANOVA, p<0.05, *post-hoc* multiple range test).

Data are means ± s.e.m. from individually calculated % DNA repair.

There was a trend in overall DNA repair capacity (% DNA repair) between species: *T. ventricosus*<*L. variegatus*<*E. l. lucunter*<*I. badionotus* (Table 2). *E. l. lucunter* and *I. badionotus* had moderate (42%) and high (71%) repair of DNA damage, respectively, 24 hours following exposure to the highest concentration of H_2_O_2_ (100 mM), and high (53%) and very high (80%) repair of DNA damage, respectively, 24 hours following exposure to the highest dose of UV-C (9999 J/m^2^); these values contrast with low (<25%) repair in *L. variegatus* and *T. ventricosus* for the highest levels of both UV-C and H_2_O_2_. *I. badionotus* had high or very high DNA repair at all levels of exposure, and *E. l. lucunter* had high or very high levels of DNA repair after exposure to concentrations of H_2_O_2_ up to 10 mM. *T. ventricosus* had moderate or low DNA repair at all levels of exposure, except 0.1 mM H_2_O_2_ (59%), and both *T. ventricosus* and *L. variegatus* had reduced DNA repair at high concentrations or doses. There was an indication among all species for higher DNA repair capacity for H_2_O_2_-induced DNA damage, compared with UV-C-induced DNA damage.

## Discussion

The objective of this study was to comparatively evaluate DNA damage and DNA repair capabilities of coelomocytes from four echinoderm species (*L. variegatus*, *E. l. lucunter*, *I. badionotus*, and *T. ventricosus*). Investigating DNA damage and DNA repair in cells of these different species can provide information on the value of coelomocytes as bioindicator cells and increase understanding of the overall impacts of genotoxicants on these organisms. Coelomocytes were chosen to evaluate the response to DNA damaging agents because they are well characterized cells involved in immunity and wound healing that have been proposed to be sensitive indicator cells for environmental stress [9], [35]–[38], [43]–[44], yet little is known of their response to genotoxicants. The coelomocyte populations differed between species with *E. l. lucunter* and *I. badionotus* having higher cell concentrations than *L. variegatus* and *T. ventricosus*. There were no differences in the percentage of red spherule cells in the coelomocytes of the three sea urchin species; however, no red spherule cells were identified in the coelomic fluid of *I. badionotus*. This is consistent with a study on the sea cucumber *Apostichopus japonicus* which identified six cell types, none of which were red spherule cells [49]. Because little is known about the DNA repair capacity of various coelomocyte types, it is unknown whether differences in composition of coelomic fluid among species play a role in the ability for coelomocytes to repair damaged DNA. In addition, differences in coelomocyte composition between individuals may be a potential source of inter-individual variations observed in both treatment groups, in particular for *I. badionotus*. Apoptosis has been reported in sea urchin embryos exposed to UV radiation [26], but high coelomocyte viability 24 hours post-exposure over the course of this study suggests that apoptosis was not a factor contributing to the levels of DNA damage. This is consistent with the report of low levels of apoptosis in coelomocytes of the sea urchin *P. lividus* exposed to up to 2000 J/m^2^ of UV-B (312 nm) [9]. Despite little cell death, 24 hours after exposure to the highest levels of H_2_O_2_ and UV-C, coelomocytes were observed to be smaller in size. A study on cultured mouse myotubes found that 24 hours of chronic exposure to H_2_O_2_ significantly reduced myotube diameter *in vitro* [50]; however, it is unknown whether this decrease in cell size may have an impact on DNA repair activity in the nucleus.

In this study, DNA damage was detected by the Fast Micromethod, as recommended for high-throughput genotoxic analyses [13] and comparable with the comet assay for DNA strand break detection and sensitivity [48]. There was a clear concentration- and dose-dependent increase in DNA damage for all echinoderm species tested. DNA damage levels in coelomocytes from *I. badionotus* appeared to be much lower than those for the sea urchin species; however, CMFSW blanks (not CFCF blanks) were subtracted from *I. badionotus* samples due to high relative fluorescent units in the CFCF from this species, which may underestimate the amount of DNA damage. Further investigation is needed to determine whether differences in the overall magnitude of SSF values of *I. badionotus* reflect high genotoxicity resistance in this species, and interspecific comparisons of overall levels of DNA damage with this species are carried out with caution. Based on the response over a similar concentration range of H_2_O_2_, the sensitivities of echinoderm coelomocytes are similar to that reported for zebrafish larvae exposed to H_2_O_2_ *in vivo*, where DNA damage (as estimated by comet assay) reached a plateau in the response curve between 100–200 mM H_2_O_2_ [51]. Other marine invertebrates such as shrimp (embryo and larvae exposures) and mussels (*in vitro* haemocyte exposures) have high levels of reported DNA damage at concentrations of H_2_O_2_ below 1 mM [37], [52]–[53]. These interspecific differences highlight the need for consideration of suitable genotoxic bioindicator species. Genotoxic exposure of HeLa cells, mouse lymphoma cells, and peripheral blood mononuclear cells resulted in SSF values in a similar range to the levels of initial damage induced in coelomocytes of sea urchins [48]. However, comparable treatments of HeLa cells exposed to 1000 J/m^2^ UV-C resulted in a SSF of 1.196 [48], considerably higher than the SSFs values of 0.28, 0.38, and 0.26 from coelomocytes of *T. ventricosus, L. variegatus and E. l. lucunter*, respectively, exposed to the same dose. This is consistent with the observation that LD_50_ values for sea urchin coelomocytes (*L. variegatus*) exposed to H_2_O_2_ and UV-C are much higher than those of mammalian cells [45], [54]–[56] and suggests that echinoderm coelomocytes are generally more resistant to genotoxicity than mammalian cells.

Comparisons of SSF values and DNA repair capacity revealed clear differences between the four species after exposure to UV-C and H_2_O_2_. It is thought that shallow coastal marine species may be readily exposed to genotoxicants and therefore evolutionarily well-adapted to repair DNA damage [57]. It is clear from the present results that coelomocytes from all species were able to repair some level of DNA damage from both genotoxic treatments, resulting in reduction in DNA damage levels within 24 hours. The time profile and temporal delay in reduction of SSF within the first 6 hours of recovery could be indicative of direct DNA repair activity as both NER and BER pathways involve removal of a nucleotide or base which temporarily produces a single-strand break in the DNA [11], [14]. The lower levels of DNA damage and pattern of a peak in DNA damage 1–6 hours after acute exposure to UV-C might indicate the relative lack of direct DNA strand breaks induced initially by UV-C exposure, and NER-induced strand breaks during repair [11], [58]–[59]. Clear indication of DNA repair in coelomocytes indicates that these cells are active in the DNA damage response system of echinoderms and supports the need for further studies of the biology of these cells.

Variability after 24 hours recovery between the four species in the present study highlights important differences in DNA repair capacity even among species that share similar habitats and presumably similar exposure to genotoxicants. Sediment-dwelling species including sea cucumbers are thought to be more susceptible to genotoxicant exposure due to direct contact with the sediment [57]; however, the results of *I. badionotus* coelomocytes indicated the species was the most effective of the selected species in DNA repair, with very high repair of DNA damage after 24 hours of recovery. Phylogenetic relationships among the echinoderms reveal *T. ventricosus* and *L. variegatus* to belong to the family Toxopneustidea, whereas, *E. l. lucunter* belongs to the family Echinometridea; both families belong to the class Echinoidea [60]. All four echinoderm species belong to the same subphylum Echinozoa. Because *T. ventricosus* and *L. variegatus* are more closely related to each other than to *E. l. lucunter*, and even less so to *I. badionotus*, it is striking that there are differences in DNA repair capacity between the two, suggesting factors more significant than ancestry are involved in determining repair capacity. One determining factor may be the lifespan of the species, and the four echinoderm species included in this study vary in their natural lifespan. Life history data indicate that *T. ventricosus* and *L. variegatus* are relatively short-lived species (<4 years) [27], [30] while *E. l. lucunter* is a longer-lived species with an estimated maximum lifespan of approximately 50 years [61]. There are very few studies of life history traits of sea cucumbers and no specific information is available for *I. badionotus* growth, survival, and longevity. However, growth data of other sea cucumber species suggest that sea cucumbers are slow-growing and long-lived. It is estimated that C*ucumaria frondosa* may take more than 25 years to reach a harvestable size [62] and modeled growth of *Holothuria nobilis* suggests that it may live for several decades [63]. DNA repair capacity (% DNA repair) after H_2_O_2_ exposure was greater in *E. l. lucunter* and *L. variegatus* than in the shorter-lived *T. ventricosus*. Additionally, percentage repair of UV-C-induced DNA damage indicated greater repair in the longer-lived *E. l. lucunter* group than in both other shorter-lived sea urchin species. A link between longevity and resistance to genotoxic stress has also been shown in bivalves with varying natural lifespans [64]–[65], and a greater repair capacity in longer-lived sea urchin species supports the idea that longer-lived species invest greater energy in cellular maintenance and repair [66]–[67]. Lack of lifespan information for *I. badionotus* restricts comparison between the species with regards to lifespan, but their highly efficient DNA repair capacity supports the speculation that they may be relatively long-lived in concordance with other sea cucumber species [62]–[63].

In conclusion, coelomocytes from different echinoderm species showed distinct differences in their sensitivity to DNA-damaging agents and their ability to repair damaged DNA over a 24-hour recovery period, therefore the choice of a single ‘sensitive’ species for ecotoxicological studies must be made with caution and consideration of differences within and between species. It is clear that coelomocytes from all species tested show some capacity for DNA repair, indicating involvement of these cells in the DNA damage response system of echinoderms; these results warrant further investigation into the biology of the DNA damage response and immune cell system in echinoderms. There was a trend for longer-lived echinoderms to have a greater DNA repair capacity compared with shorter-lived species, and it would be interesting to investigate this further with more species over a great range of natural life spans. Complete DNA repair after 24 hours recovery from exposure to both H_2_O_2_ and UV-C was evident for *I. badionotus*, while *T. ventricosus* (with the shortest estimated lifespan) had the lowest overall capacity for DNA repair. Interspecific variability in echinoderms, however, must be taken into account when considering suitable model organisms for ecotoxicological investigations, and life history characteristics such as longevity may be important determinants for species vulnerability to environmental genotoxicity.

## Supporting Information

File S1 **Supplementary data.**
(PDF)
