# Supplementary material for: Comparative DNA Damage and Repair in Echinoderm Coelomocytes Exposed to Genotoxicants
Source: PLoS One. 2014 Sep 17;9(9):e107815. doi: 10.1371/journal.pone.0107815 (PMC4168213; doi:10.1371/journal.pone.0107815)
Supplement: File S1 — Supplementary data. (PDF) [file pone.0107815.s001.pdf]

Comparative DNA Damage and Repair in Echinoderm Coelomocytes Exposed to Genotoxicants

Ameena H. El-Bibany, Andrea G. Bodnar, Helena C. Reinardy*

Molecular Discovery Laboratory, Bermuda Institute of Ocean Sciences, 17 Biological Station, St. George's, GE 01 Bermuda.

*Corresponding author. Molecular Discovery Laboratory, Bermuda Institute of Ocean Sciences, 17 Biological Station, St. George's, GE 01 Bermuda. Tel: +441 297 1880 x725. Fax: +441 297 8143.

helena.reinardy@bios.edu

SUPPLEMENTARY MATERIAL

Contents:

Table 1: Individual size, cell counts, and cell viability in all echinoderms tested. 3

Table 2: Strand scission factors (SSF) in *Lytechinus variegatus* coelomocytes over a 24-h period of recovery after acute exposure to UV-C or H_2O_2. Combined data from n=12 individuals. 4

Table 3: Strand scission factors (SSF) in animal AE2 coelomocytes over a 24-h period of recovery after acute exposure to UV-C or H_2O_2. Mean and standard error of n=2-4 technical replicates. 5

Table 4: Strand scission factors (SSF) in animal AE3 coelomocytes over a 24-h period of recovery after acute exposure to UV-C or H_2O_2. Mean and standard error of n=2-4 technical replicates. 5

Table 5: Strand scission factors (SSF) in animal AE4 coelomocytes over a 24-h period of recovery after acute exposure to UV-C or H_2O_2. Mean and standard error of n=2-4 technical replicates. 6

Table 6: Strand scission factors (SSF) in animal AE5 coelomocytes over a 24-h period of recovery after acute exposure to UV-C or H_2O_2. Mean and standard error of n=2-4 technical replicates. 6

Table 7: Strand scission factors (SSF) in animal AE6 coelomocytes over a 24-h period of recovery after acute exposure to UV-C or H_2O_2. Mean and standard error of n=2-4 technical replicates. 7

Table 8: Strand scission factors (SSF) in animal AE7 coelomocytes over a 24-h period of recovery after acute exposure to UV-C or H_2O_2. Mean and standard error of n=2-4 technical replicates. 7

Table 9: Strand scission factors (SSF) in animal AE9 coelomocytes over a 24-h period of recovery after acute exposure to UV-C or H_2O_2. Mean and standard error of n=2-4 technical replicates. 8

Table 10: Strand scission factors (SSF) in animal AE10 coelomocytes over a 24-h period of recovery after acute exposure to UV-C or H_2O_2. Mean and standard error of n=2-4 technical replicates. 8

Table 11: Strand scission factors (SSF) in animal AE11 coelomocytes over a 24-h period of recovery after acute exposure to UV-C or H_2O_2. Mean and standard error of n=2-4 technical replicates. 9

Table 12: Strand scission factors (SSF) in animal AE12 coelomocytes over a 24-h period of recovery after acute exposure to UV-C or H_2O_2. Mean and standard error of n=2-4 technical replicates. 9

Table 13: Strand scission factors (SSF) in animal AE13 coelomocytes over a 24-h period of recovery after acute exposure to UV-C or H_2O_2. Mean and standard error of n=2-4 technical replicates. 10

Table 14: Strand scission factors (SSF) in animal AE14 coelomocytes over a 24-h period of recovery after acute exposure to UV-C or H_2O_2. Mean and standard error of n=2-4 technical replicates. 10

Table 15: Strand scission factors (SSF) in *Tripneustes ventricosus* coelomocytes over a 24-h recovery period after acute exposure to UV-C or H_2O_2. Mean data from n=5 individuals. 11

Table 16: Strand scission factors (SSF) in animal AE23 coelomocytes over a 24-h period of recovery after acute exposure to UV-C or H_2O_2. Mean and standard error of n=2-4 technical replicates. 11
Table 17: Strand scission factors (SSF) in animal AE24 coelomocytes over a 24-h period of recovery after acute exposure to UV-C or H_2O_2. Mean and standard error of n=2-4 technical replicates. 12
Table 18: Strand scission factors (SSF) in animal AE25 coelomocytes over a 24-h period of recovery after acute exposure to UV-C or H_2O_2. Mean and standard error of n=2-4 technical replicates. 12
Table 19: Strand scission factors (SSF) in animal AE25 coelomocytes over a 24-h period of recovery after acute exposure to UV-C or H_2O_2. Mean and standard error of n=2-4 technical replicates. 13
Table 20: Strand scission factors (SSF) in animal AE27 coelomocytes over a 24-h period of recovery after acute exposure to UV-C or H_2O_2. Mean and standard error of n=2-4 technical replicates. 13
Table 21: Strand scission factors (SSF) in *Echinometra lucunter lucunter* coelomocytes over a 24-h period of recovery after acute exposure to UV-C or H_2O_2. Combined data from n=7 (UV-C) or n=8 (H_2O_2) individuals. 14
Table 22: Strand scission factors (SSF) in animal AE28 coelomocytes over a 24-h period of recovery after acute exposure to UV-C or H_2O_2. Mean and standard error of n=2-4 technical replicates. 14
Table 23: Strand scission factors (SSF) in animal AE29 coelomocytes over a 24-h period of recovery after acute exposure to UV-C or H_2O_2. Mean and standard error of n=2-4 technical replicates. 15
Table 24: Strand scission factors (SSF) in animal AE30 coelomocytes over a 24-h period of recovery after acute exposure to UV-C or H_2O_2. Mean and standard error of n=2-4 technical replicates. 15
Table 25: Strand scission factors (SSF) in animal AE31 coelomocytes over a 24-h period of recovery after acute exposure to UV-C or H_2O_2. Mean and standard error of n=2-4 technical replicates. 16
Table 26: Strand scission factors (SSF) in animal AE32 coelomocytes over a 24-h period of recovery after acute exposure to UV-C or H_2O_2. Mean and standard error of n=2-4 technical replicates. 16
Table 27: Strand scission factors (SSF) in animal AE33 coelomocytes over a 24-h period of recovery after acute exposure to UV-C or H_2O_2. Mean and standard error of n=2-4 technical replicates. 17
Table 28: Strand scission factors (SSF) in animal AE34 coelomocytes over a 24-h period of recovery after acute exposure to UV-C or H_2O_2. Mean and standard error of n=2-4 technical replicates. 17
Table 29: Strand scission factors (SSF) in animal AE35 coelomocytes over a 24-h period of recovery after acute exposure to UV-C or H_2O_2. Mean and standard error of n=2-4 technical replicates. 18
Table 30: Strand scission factors (SSF) in *Isostichopus badionotus* coelomocytes over a 24-h period of recovery after acute exposure to UV-C or H_2O_2. Combined data from n=8 individuals. 18
Table 31: Strand scission factors (SSF) in animal AE15 coelomocytes over a 24-h period of recovery after acute exposure to UV-C or H_2O_2. Mean and standard error of n=2-4 technical replicates. 19
Table 32: Strand scission factors (SSF) in animal AE16 coelomocytes over a 24-h period of recovery after acute exposure to UV-C or H_2O_2. Mean and standard error of n=2-4 technical replicates. 19
Table 33: Strand scission factors (SSF) in animal AE17 coelomocytes over a 24-h period of recovery after acute exposure to UV-C or H_2O_2. Mean and standard error of n=2-4 technical replicates. 20
Table 34: Strand scission factors (SSF) in animal AE18 coelomocytes over a 24-h period of recovery after acute exposure to UV-C or H_2O_2. Mean and standard error of n=2-4 technical replicates. 20
Table 35: Strand scission factors (SSF) in animal AE19 coelomocytes over a 24-h period of recovery after acute exposure to UV-C or H_2O_2. Mean and standard error of n=2-4 technical replicates. 21
Table 36: Strand scission factors (SSF) in animal AE20 coelomocytes over a 24-h period of recovery after acute exposure to UV-C or H_2O_2. Mean and standard error of n=2-4 technical replicates. 21
Table 37: Strand scission factors (SSF) in animal AE21 coelomocytes over a 24-h period of recovery after acute exposure to UV-C or H_2O_2. Mean and standard error of n=2-4 technical replicates. 22
Table 38: Strand scission factors (SSF) in animal AE22 coelomocytes over a 24-h period of recovery after acute exposure to UV-C or H_2O_2. Mean and standard error of n=2-4 technical replicates. 22

Table 1: Individual size, cell counts, and cell viability in all echinoderms tested.

Animal code	Species	Test diameter (mm)	Cell concentration (cells/µl)	Red cells (%)	Cell viability after 24-hr recovery to 9999 J/m^2 UV-C (%)	Cell viability after 24-hr recovery to 100 mM H_2O_2 (%)
**AE2**	*Lytechinus variegatus*	68	644	19.57	96.8	98.48
**AE3**		78	1684	3.92	-	-
**AE4**		53	3227	5.27	94.41	76.81
**AE5**		75	2888	14.82	99.2	99.8
**AE6**		54	1660	6.02	99.23	93.15
**AE7**		79	2273	8.8	99.03	98.91
**AE9**		54	2115	2.77	99.05	97.74
**AE10**		85	2463	9.44	95.92	99.55
**AE11**		52	1420	10.7	98.61	98.76
**AE12**		55	2558	7.82	99.28	99.19
**AE13**		71	1058	17.39	99.26	100
**AE14**		53	1500	2.17	99.8	98.15
**AE23**	*Tripneustes ventricosus*	112	2543	9.14	94.72	99.4
**AE24**		105	1900	2.11	96.54	100
**AE25**		107	1600	7.5	94.67	99.51
**AE26**		115	2290	3.49	97.26	99.22
**AE27**		101	940	15.96	90.18	99.29
**AE28**	*Echinometra lucunter lucunter*	44	1260	0	99.43	100
**AE29**		67	2990	5.69	99.68	99.15
**AE30**		71	6520	3.53	99.84	99.34
**AE31**		59	7930	7.57	99.8	100
**AE32**		40	3230	4.33	97.58	99.43
**AE33**		58	4630	28.51	99.84	100
**AE34**		68	5450	7.71	99.75	100
**AE35**		27	4510	6.43	-	100
		**Length (mean of multiple measurements, mm)**				
**AE15**	*Isostichopus badionotus*	88	9220	0	99.03	99.12
**AE16**		285	5510	0	98.68	98.92
**AE17**		159	2170	0	99.62	97.98
**AE18**		258	3160	0	100	96.65
**AE19**		150	5990	0	100	97.84
**AE20**		180	2770	0	98.73	98.43
**AE21**		149	3050	0	99.75	100
**AE22**		280	3220	0	97.88	95.95

Table 2: Strand scission factors (SSF) in *Lytechinus variegatus* coelomocytes over a 24-h period of recovery after acute exposure to UV-C or H_2O_2. Combined data from n=12 individuals.

	UV (J/m^2)									
	0		250		1000		3000		9999	
Recovery time (h)	mean	s.e.m.	mean	s.e.m.	mean	s.e.m.	mean	s.e.m.	mean	s.e.m.
0	0.022945	0.01141	0.215311	0.031829	0.382904	0.04611	0.540263	0.042626	0.553551	0.0414
1	0.008955	0.002649	0.137037	0.059959	0.370643	0.033001	0.393845	0.054593	0.49242	0.026797
3	0.013324	0.006952	0.333903	0.074731	0.379989	0.037191	0.640131	0.066259	0.733059	0.095821
6	0.013012	0.005692	0.160645	0.031466	0.232612	0.034092	0.475246	0.046055	0.637588	0.05538
24	0.006925	0.001662	0.174892	0.040573	0.379542	0.030992	0.502716	0.029378	0.591843	0.041555
	H_2O_2 (mM)									
	0		0.1		1		10		100 mM	
Recovery time (h)	mean	s.e.m.	mean	s.e.m.	mean	s.e.m.	mean	s.e.m.	mean	s.e.m.
0	0.012872	0.005294	0.390092	0.044918	0.574944	0.072064	0.736172	0.058181	0.780006	0.054118
1	0.028128	0.011459	0.279413	0.049947	0.512917	0.05048	0.546464	0.072976	0.668603	0.071449
3	0.006839	0.002163	0.164511	0.04519	0.454423	0.058838	0.577209	0.086963	0.739822	0.068437
6	-0.01716	0.021741	0.152054	0.035277	0.255411	0.042387	0.405147	0.075322	0.756255	0.081491
24	0.008852	0.00375	0.046133	0.023586	0.128078	0.035133	0.235723	0.045413	0.668079	0.10433

Table 3: Strand scission factors (SSF) in animal AE2 coelomocytes over a 24-h period of recovery after acute exposure to UV-C or H_2O_2. Mean and standard error of n=2-4 technical replicates.

**AE2** *L. variegatus*	UV (J/m^2)									
	0		250		1000		3000		9999	
Recovery time (h)	mean	s.e.m.	mean	s.e.m.	mean	s.e.m.	mean	s.e.m.	mean	s.e.m.
0	0.007467	0.045134	0.191462	0.107072	0.374575	0.051952	0.645342	0.114358	0.638121	0.060378
1	0.003089	0.051863	-0.0725	0.048636	0.220946	0	0.60459	0.081188	0.58148	0.130654
3	0.004991	0.046427	0.137799	0.222502	0.344015	0.024095	0.741261	0.113417	1.006851	0.119251
6	0.015847	0.081727	0.216687	0.088544	0.431567	0.089969	0.799617	0.116708	0.868636	0.055797
24	0.000716	0.020366	0.08915	0.079261	0.272704	0.022526	0.486375	0.022744	0.818886	0.036693
	H_2O_2 (mM)									
	0		0.1		1		10		100	
Recovery time (h)	mean	s.e.m.	mean	s.e.m.	mean	s.e.m.	mean	s.e.m.	mean	s.e.m.
0	0.002845	0.049766	0.331457	0.066065	0.816241	0.154382	1.134483	0.16481	0.926279	0.115384
1	0.010477	0.058635	0.228621	0.066164	0.444514	0.051952	0.834316	0.102341	0.733617	0.046547
3	0.024645	0.100886	0.358165	0.281513	0.634346	0.323777	0.779601	0.099141	0.815561	0.1641
6	0.015354	0.083035	0.055112	0.069415	0.256156	0.077573	0.142943	0.271909	0.687896	0.052808
24	0.005923	0.050296	-0.04294	0.030139	0.03613	0.050053	0.110863	0.051952	0.715654	0.025079

Table 4: Strand scission factors (SSF) in animal AE3 coelomocytes over a 24-h period of recovery after acute exposure to UV-C or H_2O_2. Mean and standard error of n=2-4 technical replicates.

**AE3** *L. variegatus*	UV (J/m^2)									
	0		250		1000		3000		9999	
Recovery time (h)	mean	s.e.m.	mean	s.e.m.	mean	s.e.m.	mean	s.e.m.	mean	s.e.m.
0	0.052497	0.217864	-0.02292	0.146654						
1	0.010047	0.068035	-0.28289	0.311844	0.471607	0.218251			0.567229	0.36858
3	0.085746	0.194119	0.558586	0.020136	0.63152	0.189476	1.183835	0.060701	1.558017	0.050761
6	0.020149	0.09722	0.46688	0.322191	0.261983	0.100073	0.618489	0.119372	0.882902	0.303715
24	6.27E-05	0.005216	0.062641	0.124851	0.313956	0.057861	0.553192	0.070614	0.69093	0.014086
	H_2O_2 (mM)									
	0		0.1		1		10		100	
Recovery time (h)	mean	s.e.m.	mean	s.e.m.	mean	s.e.m.	mean	s.e.m.	mean	s.e.m.
0	0.059047	0.16275								
1	0.142347	0.287259	-0.17034	0.162839	0.33031	0.050852	0.50978	0.072996	0.694125	0.207793
3	0.01689	0.087013	0.011562	0.056815	0.517224	0.02402	0.830096	0.173499	1.027731	0.184287
6	0.001657	0.026636	-0.03133	0.088175	0.147223	0.104098	0.321421	0.121198	0.543335	0.128538
24	0.040884	0.130093	0.055606	0.097564	0.254644	0.089714	0.487278	0.094748	0.834488	0.045312

Table 5: Strand scission factors (SSF) in animal AE4 coelomocytes over a 24-h period of recovery after acute exposure to UV-C or H_2O_2. Mean and standard error of n=2-4 technical replicates.

**AE4** *L. variegatus*	UV (J/m^2) 0		250		1000		3000		9999	
Recovery time (h)	mean	s.e.m.	mean	s.e.m.	mean	s.e.m.	mean	s.e.m.	mean	s.e.m.
0	0.140175	0.429014	0.382227	0.149021	0.449633	0.242432	0.68933	0.302275	0.539813	0.10487
1	0.034776	0.200384	0.230021	0.148948	0.431501	0.21246	-0.07261	0.207759	0.36766	0.180113
3	0.001777	0.027988	0.103785	0.068674	0.253601	0.061892	0.413234	0.058524	0.641919	0.015069
6	0.001491	0.036011	0.049895	0.11099	0.105743	0.094707	0.332932	0.044787	0.579054	0.098498
24	0.002607	0.033744	0.22461	0.031454	0.411025	0.086526	0.466135	0.023691	0.679725	0.060469
	H_2O_2 (mM) 0		0.1		1		10		100	
Recovery time (h)	mean	s.e.m.	mean	s.e.m.	mean	s.e.m.	mean	s.e.m.	mean	s.e.m.
0	0.038432	0.137623	0.301495	0.130456	0.590017	0.081402	0.534879	0.297831	0.633584	0.154258
1	0.017992	0.091383	0.219039	0.174394	0.392594	0.068525	0.272111	0.189053	0.349263	0.216449
3	0.000168	0.14343	-0.12011	0.027003	0.186179	0.05403	0.155315	0.033388	0.692713	0.092776
6	-1.7E-07	0	0.08257	0.102876	0.087363	0.058059	0.239622	0.213708	0.519983	0.040743
24	0.00472	0.064146	0	0.046513	0.105842	0.029434	0.102997	0.044348	0.48659	0.014878

Table 6: Strand scission factors (SSF) in animal AE5 coelomocytes over a 24-h period of recovery after acute exposure to UV-C or H_2O_2. Mean and standard error of n=2-4 technical replicates.

**AE5** *L. variegatus*	UV (J/m^2) 0		250		1000		3000		9999	
Recovery time (h)	mean	s.e.m.	mean	s.e.m.	mean	s.e.m.	mean	s.e.m.	mean	s.e.m.
0	0.009284	0.062486	0.249072	0.072825	0.258217	0.047009	0.430597	0.078864	0.619042	0.05389
1	0.005995	0.051789	0.397034	0.047635	0.467605	0.012674	0.579841	0.049358	0.670199	0.042292
3	0.00099	0.020584	0.293925	0.055341	0.332763	0.07891	0.600442	0.039953	0.865749	0.018889
6	0.002639	0.03346	0.153307	0.05006	0.214941	0.013714	0.347593	0.12516	0.683182	0.005637
24	0.010087	0.066428	0.25555	0.107588	0.289114	0.096158	0.457615	0.098391	0.545987	0.041635
	H_2O_2 (mM) 0		0.1		1		10		100	
Recovery time (h)	mean	s.e.m.	mean	s.e.m.	mean	s.e.m.	mean	s.e.m.	mean	s.e.m.
0	0.001224	0.023243	0.315301	0.063702	0.563165	0.043879	0.723951	0.049046	0.867686	0.019179
1	0.048627	0.142832	0.34502	0.049604	0.637739	0.030408	0.829586	0.092154	0.979078	0.039273
3	0.000537	0.015244	0.096054	0.083725	0.24803	0.052367	0.502273	0.087227	0.480277	0.301507
6	0.002366	0.031696	0.193949	0.170465	0.162727	0.021474	0.235219	0.049212	0.59817	0.056118
24	0.000535	0.021576	-0.10961	0.050509	-0.1175	0.050266	0.038065	0.101578	0.248743	0.059624

Table 7: Strand scission factors (SSF) in animal AE6 coelomocytes over a 24-h period of recovery after acute exposure to UV-C or H_2O_2. Mean and standard error of n=2-4 technical replicates.

**AE6** *L. variegatus*	UV (J/m^2) 0		250		1000		3000		9999	
Recovery time (h)	mean	s.e.m.	mean	s.e.m.	mean	s.e.m.	mean	s.e.m.	mean	s.e.m.
0	0.024965	0.10623	0.234119	0.026462	0.378793	0.118671	0.437642	0.018494	0.504745	0.010389
1	0.000594	0.022726	0.146371	0.06364	0.2595	0.028206	0.423921	0.423921	0.468139	0.468139
3	0.009586	0.063231	0.15881	0.057982	0.320435	0.011902	0.407229	0.087321	0.438559	0.027501
6	0.001605	0.026522	0.081436	0.071001	0.111586	0.091377	0.216978	0.09325	0.334206	0.01969
24	0.003945	0.058627	0.105835	0.049097	0.218186	0.037479	0.375321	0.032455	0.432847	0.02687
	H_2O_2 (mM) 0		0.1		1		10		100	
Recovery time (h)	mean	s.e.m.	mean	s.e.m.	mean	s.e.m.	mean	s.e.m.	mean	s.e.m.
0	0.002215	0.030812	0.073495	0.023625	0.075093	0.027246	0.453147	0.094704	0.854552	0.319957
1	0.054434	0.222011	0.367938	0.167024	0.555684	0.059605	0.176703	0.576204	1.188695	0.202934
3	0.004865	0.046557	-0.01436	0.010452	0.122387	0.086044	0.191758	0.055708	0.759206	0.094972
6	0.004054	0.041641	-0.04677	0.068711	-0.04121	0.084926	-0.11884	0.055391	1.022995	0.093276
24	0.000837	0.019035	0.043705	0.061763	0	0.024576	0.143216	0.037547	0.505118	0.060433

Table 8: Strand scission factors (SSF) in animal AE7 coelomocytes over a 24-h period of recovery after acute exposure to UV-C or H_2O_2. Mean and standard error of n=2-4 technical replicates.

**AE7** *L. variegatus*	UV (J/m^2) 0		250		1000		3000		9999	
Recovery time (h)	mean	s.e.m.	mean	s.e.m.	mean	s.e.m.	mean	s.e.m.	mean	s.e.m.
0	0.006733	0.055342	0.149238	0.084211	0.146822	0.013319	0.358398	0.015036	0.364037	0.042216
1	0.009754	0.066883	0.255262	0.066756	0.298883	0.036234	0.336919	0.068384	0.415654	0.067158
3	0.011117	0.070158	0.154429	0.046839	0.249342	0.088863	0.367574	0.092163	0.478505	0.053397
6	0.01258	0.072295	0.12453	0.041508	0.124887	0.023505	0.375935	0.051082	0.532884	0.001171
24	0.000153	0.008165	0.063021	0.089174	0.292033	0.082108	0.273971	0.052702	0.274029	0.020345
	H_2O_2 (mM) 0		0.1		1		10		100	
Recovery time (h)	mean	s.e.m.	mean	s.e.m.	mean	s.e.m.	mean	s.e.m.	mean	s.e.m.
0	0.002785	0.03436	0.438089	0.099348	0.23595	0.233159	0.615162	0.041725	0.645374	0.091771
1	0.010231	0.068102	0.182765	0.035362	0.228361	0.021692	0.32523	0.051054	0.395192	0.029702
3	0.003204	0.037475	0.088785	0.19545	0.213889	0.079268	0.228107	0.03758	0.300461	0.006155
6	0.002561	0.033124	0.218231	0.083009	0.336019	0.038535	0.432468	0.045833	0.580961	0.041979
24	0.004481	0.043464	0.069151	0.032447	0.082048	0.043643	0.194097	0.050597	0.269967	0.040855

Table 9: Strand scission factors (SSF) in animal AE9 coelomocytes over a 24-h period of recovery after acute exposure to UV-C or H_2O_2. Mean and standard error of n=2-4 technical replicates.

**AE9** *L. variegatus*	UV (J/m^2) 0		250		1000		3000		9999	
Recovery time (h)	mean	s.e.m.	mean	s.e.m.	mean	s.e.m.	mean	s.e.m.	mean	s.e.m.
0	0.001438	0.02516	0.197599	0.047463	0.508791	0.015744	0.62358	0.074575	0.580108	0.03749
1	3.02E-05	0.005089	0.179373	0.004888	0.356627	0.045445	0.421265	0.052896	0.504794	0.060675
3	0.000389	0.013062	0.486388	0.190242	0.543222	0.090199	0.627956	0.122551	0.631245	0.041428
6	0.000821	0.026712	0.164596	0.01841	0.307503	0.032265	0.519278	0.066395	0.609928	0.03964
24	0.006875	0.055513	0.42114	0.343895	0.460707	0.114521	0.586792	0.067969	0.534145	0.043055
	H_2O_2 (mM) 0		0.1		1		10		100	
Recovery time (h)	mean	s.e.m.	mean	s.e.m.	mean	s.e.m.	mean	s.e.m.	mean	s.e.m.
0	0.002531	0.046928	0.659435	0.018053	0.831647	0.077695	0.958862	0.1926	0.857979	0.043564
1	0.007571	0.081328	0.449096	0.087113	0.796195	0.241711	0.731633	0.084855	0.648957	0.075265
3	0.010922	0.097811	0.195772	0.072731	0.513845	0.148989	0.334405	0.224282	0.534884	0.026001
6	0.002275	0.031437	0.255097	0.043704	0.403345	0.046155	0.731999	0.109422	1.249755	0.053624
24	0.001754	0.027334	0.144696	0.034082	0.23135	0.601219	0.484488	0.405239	0.819229	0.070836

Table 10: Strand scission factors (SSF) in animal AE10 coelomocytes over a 24-h period of recovery after acute exposure to UV-C or H_2O_2. Mean and standard error of n=2-4 technical replicates.

**AE10** *L. variegatus*	UV (J/m^2) 0		250		1000		3000		9999	
Recovery time (h)	mean	s.e.m.	mean	s.e.m.	mean	s.e.m.	mean	s.e.m.	mean	s.e.m.
0	0.002686	0.033774	0.067136	0.045144	0.375187	0.025776	0.572308	0.060861	0.556961	0.052411
1	0.012029	0.072552	-0.17941	0.296792	0.266751	0.117303	0.420522	0.03833	0.463679	0.027967
3	0.002012	0.041836	0.088132	0.07444	0.276612	0.054761	0.584976	0.076364	0.600196	0.037578
6	0.001579	0.037045	0.088794	0.063462	0.206715	0.070388	0.412723	0.053127	0.506375	0.050797
24	0.010099	0.094023	0.137141	0.181243	0.473772	0.046073	0.618625	0.037285	0.713136	0.054235
	H_2O_2 (mM) 0		0.1		1		10		100	
Recovery time (h)	mean	s.e.m.	mean	s.e.m.	mean	s.e.m.	mean	s.e.m.	mean	s.e.m.
0	0.003449	0.039337	0.377581	0.063087	0.687673	0.038122	0.830929	0.138027	0.741989	0.056966
1	0.000838	0.026978	0.292865	0.017795	0.602779	0	0.439035	0.24517	0.722585	0.028332
3	0.002029	0.029933	0.202939	0.068534	0.483026	0.033912	0.724966	0.128145	0.93112	0.123536
6	0.000819	0.018951	0.243979	0.05737	0.330524	0.099295	0.392104	0	0.61756	0.067089
24	0.002721	0.048666	0.053151	0.060878	0.164826	0.063376	0.074755	0.057107	0.540028	0.122627

Table 11: Strand scission factors (SSF) in animal AE11 coelomocytes over a 24-h period of recovery after acute exposure to UV-C or H_2O_2. Mean and standard error of n=2-4 technical replicates.

**AE11** *L. variegatus*	UV (J/m^2) 0		250		1000		3000		9999	
Recovery time (h)	mean	s.e.m.	mean	s.e.m.	mean	s.e.m.	mean	s.e.m.	mean	s.e.m.
0	0.003666	0.039925	0.257606	0.043478	0.218614	0.112523	0.396365	0.068061	0.429475	0.077761
1	0.003996	0.041683	0.177765	0.095084	0.322141	0.048424	0.417115	0.040793	0.524269	0.092827
3	0.028832	0.117589	0.250678	0.034527	0.330792	0.080711	0.726126	0.097157	0.357504	0.173489
6	0.003625	0.039146	0.118952	0.067786	0.32697	0.074693	0.510039	0.034927	0.524989	0.061031
24	0.009081	0.064542	0.251284	0.052089	0.524024	0.06279	0.630503	0.092829	0.695828	0.116616
	H_2O_2 (mM) 0		0.1		1		10		100	
Recovery time (h)	mean	s.e.m.	mean	s.e.m.	mean	s.e.m.	mean	s.e.m.	mean	s.e.m.
0	0.005148	0.048127	0.365925	0.037255	0.516405	0.048401	0.610111	0.03164	1.172919	0.19565
1	0.008524	0.060327	0.379752	0.030488	0.366495	0.071418	0.589125	0.041825	0.711781	0.032038
3	3.36E-05	0.003803	0.389924	0.061071	0.572937	0.24064	1.075383	0	0.802062	0.081977
6	0.019632	0.096396	0.370788	0.05813	0.49389	0.044191	0.778201	0.029465	0.860039	0.023399
24	0.029981	0.118663	0.090389	0.070448	0.130989	0.059202	0.287615	0.07409	0.900024	0.123449

Table 12: Strand scission factors (SSF) in animal AE12 coelomocytes over a 24-h period of recovery after acute exposure to UV-C or H_2O_2. Mean and standard error of n=2-4 technical replicates.

**AE12** *L. variegatus*	UV (J/m^2) 0		250		1000		3000		9999	
Recovery time (h)	mean	s.e.m.	mean	s.e.m.	mean	s.e.m.	mean	s.e.m.	mean	s.e.m.
0	0.01079	0.068829	0.306541	0.174	0.721832	0.131264	0.608632	0.114279	0.643622	0.110397
1	0.013396	0.078748	0.326865	0.105071	0.62947	0.03082	0.464152	0.026849	0.460575	0.076915
3	0.004682	0.045154	0.868771	0.133619	0.567787	0.019439	0.897758	0.075425	0.920315	0.176457
6	0.000199	0.00928	0.15046	0.026988	0.419697	0.088267	0.577616	0.094275	0.645785	0.069682
24	0.019627	0.094182	0.424031	0.070707	0.545012	0.077716	0.512923	0.168698	0.587519	0.060204
	H_2O_2 (mM) 0		0.1		1		10		100	
Recovery time (h)	mean	s.e.m.	mean	s.e.m.	mean	s.e.m.	mean	s.e.m.	mean	s.e.m.
0	0.003575	0.040056	0.518277	0.046406	0.745442	0.045135	0.786838	0.107107	0.563284	0.053786
1	0.012716	0.074631	0.206435	0.039436	0.412975	0.073997	0.238367	0.224535	0.333434	0.039063
3	0.005803	0.051302	0.264112	0.020728	0.64297	0.10726	0.689372	0.053658	0.822069	0.092484
6	0.000504	0.020918	0.20988	0.06296	0.352374	0.073659	0.576018	0.032732	0.8788	0.140195
24	0.000801	0.021543	0	0.047213	0.116814	0.058903	0.260987	0.16732	0.658148	0.072744

Table 13: Strand scission factors (SSF) in animal AE13 coelomocytes over a 24-h period of recovery after acute exposure to UV-C or H_2O_2. Mean and standard error of n=2-4 technical replicates.

**AE13** *L. variegatus*	UV (J/m^2) 0		250		1000		3000		9999	
Recovery time (h)	mean	s.e.m.	mean	s.e.m.	mean	s.e.m.	mean	s.e.m.	mean	s.e.m.
0	0.00434	0.043491	0.272435	0.107187	0.399543	0.079679	0.399821	0.071897	0.373369	0.050609
1	0.008064	0.059669	0.287323	0.022457	0.365622	0.014985	0.271176	0.0737	0.343553	0.031246
3	0.002863	0.035343	0.201862	0.051799	0.316736	0.022874	0.520794	0.03371	0.474638	0.060494
6	0.026667	0.112199	0.09773	0.06915	0.17816	0.040012	0.389229	0.077637	0.4862	0.125227
24	0.009001	0.061878	0.045607	0.115846	0.424283	0.134994	0.542049	0.080925	0.571469	0.143615
	H_2O_2 (mM) 0		0.1		1		10		100	
Recovery time (h)	mean	s.e.m.	mean	s.e.m.	mean	s.e.m.	mean	s.e.m.	mean	s.e.m.
0	0.009814	0.064942	0.408186	0.038235	0.508031	0.035666	0.702455	0.140235	0.589598	0.117321
1	0.004055	0.042164	0.503476	0.107136	0.679884	0.181076	0.789501	0.233937	0.581513	0.079858
3	0.004197	0.042163	0.190572	0.12497	0.670216	0.06752	0.529111	0.05911	0.581628	0.013948
6	0.0003	0.011387	0.133863	0.188778	0.270245	0.122112	0.479102	0.150631	0.336294	0.137525
24	0.002072	0.030276	0.129937	0.085088	0.327771	0.082223	0.221383	0.056935	0.439428	0.033179

Table 14: Strand scission factors (SSF) in animal AE14 coelomocytes over a 24-h period of recovery after acute exposure to UV-C or H_2O_2. Mean and standard error of n=2-4 technical replicates.

**AE14** *L. variegatus*	UV (J/m^2) 0		250		1000		3000		9999	
Recovery time (h)	mean	s.e.m.	mean	s.e.m.	mean	s.e.m.	mean	s.e.m.	mean	s.e.m.
0	0.011303	0.072267	0.299216	0.07161	0.379936	0.023447	0.780882	0.058953	0.839762	0.144075
1	0.005689	0.062105	0.179235	0.143713	0.357065	0.108925	0.465393	0.057232	0.541808	0.100304
3	0.0069	0.055955	0.703671	0.117544	0.393045	0.031218	0.610388	0.077865	0.823204	0.094803
6	0.068943	0.178107	0.214467	0.125	0.101595	0.196299	0.60252	0.207242	0.99692	0.15388
24	0.01084	0.067858	0.018691	0.053091	0.329687	0.057905	0.52909	0.096014	0.55762	0.035046
	H_2O_2 (mM) 0		0.1		1		10		100	
Recovery time (h)	mean	s.e.m.	mean	s.e.m.	mean	s.e.m.	mean	s.e.m.	mean	s.e.m.
0	0.023397	0.129237	0.501766	0.103972	0.754725	0.097401	0.747079	0.069878	0.726824	0.08918
1	0.019719	0.095335	0.348289	0.010208	0.707472	0.073891	0.822181	0.048597	0.685003	0.098407
3	0.008775	0.063434	0.310716	0.088518	0.648025	0.083504	0.886116	0.069646	1.130159	0.009767
6	-0.25547	0.314878	0.139282	0.031276	0.266283	0.674544	0.651511	0.175749	1.179277	0.159663
24	0.01151	0.072566	0.160092	0.033634	0.207237	0.064619	0.422937	0.093053	1.599524	0

Table 15: Strand scission factors (SSF) in *Tripneustes ventricosus* coelomocytes over a 24-h recovery period after acute exposure to UV-C or H_2O_2. Mean data from n=5 individuals.

	UV (J/m^2) 0		250		1000		3000		9999	
Recovery time (h)	mean	s.e.m.	mean	s.e.m.	mean	s.e.m.	mean	s.e.m.	mean	s.e.m.
0	0.021633	0.012668	0.138564	0.015073	0.286802	0.058386	0.609652	0.109626	0.779117	0.114192
1	0.029316	0.010385	0.299695	0.04897	0.563514	0.062787	0.80334	0.105446	0.824869	0.043271
3	0.015585	0.005507	0.061989	0.072417	0.332755	0.065466	0.615217	0.071503	0.760879	0.074267
6	0.008612	0.00545	0.11652	0.105434	0.303652	0.106105	0.553739	0.124109	0.763414	0.100919
24	0.006908	0.003664	0.259633	0.093056	0.519865	0.095403	0.633973	0.120819	0.681888	0.115706
	H_2O_2 (mM) 0		0.1		1		10		100	
Recovery time (h)	mean	s.e.m.	mean	s.e.m.	mean	s.e.m.	mean	s.e.m.	mean	s.e.m.
0	0.044199	0.013544	0.577326	0.08332	0.819613	0.134333	0.753104	0.100812	0.698659	0.094053
1	0.044639	0.021606	0.51656	0.054239	0.693435	0.117378	0.755352	0.119175	0.669919	0.090221
3	0.014493	0.003318	0.526375	0.064663	0.848221	0.074633	0.880382	0.121441	0.722028	0.07512
6	0.006234	0.003283	0.318486	0.06924	0.740947	0.055	0.997551	0.042382	1.337601	0.143108
24	0.014644	0.004645	0.214267	0.073053	0.601046	0.164692	0.534535	0.120979	0.624524	0.116777

Table 16: Strand scission factors (SSF) in animal AE23 coelomocytes over a 24-h period of recovery after acute exposure to UV-C or H_2O_2. Mean and standard error of n=2-4 technical replicates.

**AE23** *T. ventricosus*	UV (J/m^2) 0		250		1000		3000		9999	
Recovery time (h)	mean	s.e.m.	mean	s.e.m.	mean	s.e.m.	mean	s.e.m.	mean	s.e.m.
0	0.069987	0.16312	0.083218	0.083712	0.156819	0.107301	0.358296	0.062902	0.492419	0.028804
1	0.005797	0.041204	0.22144	0.028379	0.533124	0.069364	0.614542	0.031295	0.904364	0.118078
3	0.019999	0.07961	0.239826	0.168471	0.536268	0.133368	0.735544	0.058074	0.795081	0.090323
6	0.000149	0.011359	0.053366	0.068699	0.210362	0.119548	0.832855	0.138906	0.717446	0.134945
24	0	0	0.478098	0.120558	0.767568	0.068712	0.937259	0.146846	0.725228	0.061995
	H_2O_2 (mM) 0		0.1		1		10		100	
Recovery time (h)	mean	s.e.m.	mean	s.e.m.	mean	s.e.m.	mean	s.e.m.	mean	s.e.m.
0	0.043903	0.124273	0.394478	0.045238	0.668638	0.059797	0.584446	0.074968	0.480327	0.072489
1	0.117233	0.234999	0.551152	0.080638	0.375735	0.226706	0.606799	0.028202	0.521587	0.055148
3	0.004941	0.047233	0.36172	0.117841	0.626757	0.095081	0.434213	0.248637	0.440053	0.055728
6	0.01598	0.086519	0.261427	0.074866	0.900059	0.087357	1.02822	0.158197	0.964494	0.089254
24	0.019414	0.092084	0.278778	0	0.819169	0.108545	0.412058	0.174215	0.689426	0.042887

Table 17: Strand scission factors (SSF) in animal AE24 coelomocytes over a 24-h period of recovery after acute exposure to UV-C or H_2O_2. Mean and standard error of n=2-4 technical replicates.

**AE24** *T. ventricosus*	UV (J/m^2) 0		250		1000		3000		9999	
Recovery time (h)	mean	s.e.m.	mean	s.e.m.	mean	s.e.m.	mean	s.e.m.	mean	s.e.m.
0	0.00673	0.055346	0.16354	-0.03173	0.435849	-0.0403	0.797574	-0.13031	0.758884	0.040615
1	0.028866	0.088704	0.214276	0.085271	0.589173	0.134624	0.68001	0.125688	0.944227	0.11246
3	2.77E-05	0.004904	0.109542	0.040539	0.330999	0.118624	0.757473	0.182788	1.033822	0.079849
6	0.001152	0.031649	0.487914	0.083525	0.593771	0.218248	0.68454	0.097195	1.085731	0.139514
24	0.006364	0.074528	0.452119	0.084661	0.61141	0.24723	0.816205	0.160682	0.921787	0.362423
	H_2O_2 (mM) 0		0.1		1		10		100	
Recovery time (h)	mean	s.e.m.	mean	s.e.m.	mean	s.e.m.	mean	s.e.m.	mean	s.e.m.
0	0.048231	0.124553	0.453037	0.429479	0.947887	0.228796	0.708443	0.0456	0.476152	0.046873
1	0.013954	0.065948	0.6385	0.09353	0.962087	0.188359	0.972129	0.176669	0.628521	0.061432
3	0.00939	0.064071	0.513876	0.038789	0.980556	0.231436	1.00836	0.01438	0.710637	0.025413
6	0.003141	0.052299	0.086055	0.087712	0.562648	0.072585	1.119965	0.097341	1.698641	0.09519
24	0.007224	0.057094	0.267829	0.190565	0.877024	0.28085	0.501824	0.043938	0.392796	0.033598

Table 18: Strand scission factors (SSF) in animal AE25 coelomocytes over a 24-h period of recovery after acute exposure to UV-C or H_2O_2. Mean and standard error of n=2-4 technical replicates.

**AE25** *T. ventricosus*	UV (J/m^2) 0		250		1000		3000		9999	
Recovery time (h)	mean	s.e.m.	mean	s.e.m.	mean	s.e.m.	mean	s.e.m.	mean	s.e.m.
0	0.00367	0.033442	0.137958	0.062667	0.245709	0.073179	0.887845	0.249116	0.637953	0.081955
1	0.030336	0.108774	0.310095	0.007565	0.497976	0.035557	1.193604	0.278602	0.802092	0.02801
3	0.015318	0.064718	0.153764	0.023076	0.250137	0.027857	0.698047	0.072466	0.697839	0.042465
6	0.014094	0.111242	0.192769	0.060517	0.439644	0.122973	0.667381	0.072	0.823117	0.063778
24	0.001062	0.021374	0.19422	0.044892	0.486055	0.033693	0.664533	0.056455	0.700018	0.065965
	H_2O_2 (mM) 0		0.1		1		10		100	
Recovery time (h)	mean	s.e.m.	mean	s.e.m.	mean	s.e.m.	mean	s.e.m.	mean	s.e.m.
0	0.031699	0.098945	0.816569	0.170373	1.229943	0.372516	1.029154	0.160761	0.937628	0.151895
1	0.070919	0.144414	0.420777	0.127252	0.861684	0.257564	0.859399	0.072918	0.746335	0.075029
3	0.021933	0.079114	0.737891	0.018503	1.040496	0.195817	1.106985	0.048087	0.845963	0.010385
6	0.012025	0.074062	0.3796	0.050805	0.732906	0.111231	1.032468	0.092047	1.175807	0.164789
24	0.023969	0.081614	0.253632	0.084099	0.478803	0.033642	0.90991	0.04028	1.01823	0.059231

Table 19: Strand scission factors (SSF) in animal AE25 coelomocytes over a 24-h period of recovery after acute exposure to UV-C or H_2O_2. Mean and standard error of n=2-4 technical replicates.

**AE26** *T. ventricosus*	UV (J/m²) 0		250		1000		3000		9999	
Recovery time (h)	mean	s.e.m.	mean	s.e.m.	mean	s.e.m.	mean	s.e.m.	mean	s.e.m.
0	0.003721	0.032806	0.167776	0.049109	0.181331	0.157759	0.646163	0.037528	0.833066	0.028458
1	0.014915	0.065929	0.269828	0.039901	0.410902	0.011153	0.854176	0.118455	0.757577	0.048593
3	0.033119	0.101423	-0.17494	0.013424	0.149955	0.082124	0.419898	0.047993	0.659747	0.036348
6	0.027656	0.156639	-0.09407	0.014654	0.308143	0	0.469547	0.073408	0.732616	0.184616
24	0.020555	0.09627	0.19739	0.133945	0.546743	0.145819	0.503705	0.164575	0.816323	0.180016
	H_2O_2 (mM) 0		0.1		1		10		100	
Recovery time (h)	mean	s.e.m.	mean	s.e.m.	mean	s.e.m.	mean	s.e.m.	mean	s.e.m.
0	0.007003	0.046652	0.734604	0.178052	0.823486	0.058374	0.939282	0.028384	0.833192	0.068635
1	0.003147	0.030489	0.612185	0.107191	0.816912	0.166881	0.976893	0.11174	0.978391	0.052328
3	0.014825	0.063142	0.583451	0.181315	0.832373	0.170367	0.816696	0.135109	0.764862	0.091903
6	2.28E-05	0.004454	0.498971	0.138684	0.716106	0.24597	0.880013	0.142728	1.646732	0.215523
24	0.000252	0.01481	0.342623	0.111263	0.824169	0.059391	0.660555	0.27749	0.641632	0.112448

Table 20: Strand scission factors (SSF) in animal AE27 coelomocytes over a 24-h period of recovery after acute exposure to UV-C or H_2O_2. Mean and standard error of n=2-4 technical replicates.

**AE27** *T. ventricosus*	UV (J/m²) 0		250		1000		3000		9999	
Recovery time (h)	mean	s.e.m.	mean	s.e.m.	mean	s.e.m.	mean	s.e.m.	mean	s.e.m.
0	0.024055	0.105006	0.140329	0.050426	0.414301	0.135654	0.358381	0.084759	1.173264	0.468036
1	0.066665	0.157962	0.482838	0.173057	0.786397	0.333649	0.674369	0.083388	0.716083	0.269574
3	0.009461	0.050572	-0.01825	0.024143	0.396418	0.026129	0.465124	0.089203	0.617905	0.008737
6	7.64E-06	0.001823	-0.05738	0.062689	-0.03366	0.049709	0.114371	0.047935	0.458159	0.045626
24	0.006558	0.052768	-0.02366	0.09808	0.187551	0.093057	0.248163	0.069438	0.246083	0.053886
	H_2O_2 (mM) 0		0.1		1		10		100	
Recovery time (h)	mean	s.e.m.	mean	s.e.m.	mean	s.e.m.	mean	s.e.m.	mean	s.e.m.
0	0.090162	0.186533	0.48794	0.093108	0.42811	0.01712	0.504193	0	0.765996	0.117014
1	0.017941	0.074887	0.360188	0.200339	0.450759	0.216202	0.36154	0.073836	0.474762	0
3	0.021375	0.09998	0.434935	0.046173	0.760925	0.066475	1.035657	0.129662	0.848625	0.017621
6	0	0	0.366379	0.106305	0.793016	0.064472	0.927089	0.076288	1.202332	0
24	0.022361	0.140563	-0.07153	0.088121	0.006067	0.164622	0.188328	0.068709	0.380535	0.013559

Table 21: Strand scission factors (SSF) in *Echinometra lucunter lucunter* coelomocytes over a 24-h period of recovery after acute exposure to UV-C or H_2O_2. Combined data from n=7 (UV-C) or n=8 (H_2O_2) individuals.

	UV (J/m^2) 0		250		1000		3000		9999	
Recovery time (h)	mean	s.e.m.	mean	s.e.m.	mean	s.e.m.	mean	s.e.m.	mean	s.e.m.
0	0.039912	0.017086	0.157756	0.072205	0.267489	0.152496	0.273463	0.102138	0.595563	0.243574
1	0.02074	0.007508	0.130966	0.058269	0.309691	0.093221	0.413006	0.133476	0.652577	0.149268
3	0.024677	0.009576	0.199109	0.047925	0.303049	0.145637	0.331392	0.145441	0.695142	0.277647
6	0.100714	0.078714	0.154496	0.044166	0.327636	0.19081	0.302351	0.241981	0.699348	0.173589
24	0.00933	0.003461	0.283598	0.085723	0.238785	0.055528	0.344514	0.133721	0.381015	0.174629
	H_2O_2 (mM) 0		0.1		1		10		100	
Recovery time (h)	mean	s.e.m.	mean	s.e.m.	mean	s.e.m.	mean	s.e.m.	mean	s.e.m.
0	0.015725	0.006671	0.176222	0.063877	0.550062	0.07267	1.01632	0.127514	0.897583	0.060978
1	0.026654	0.009514	0.135023	0.06465	0.445645	0.109801	0.72749	0.112318	0.836998	0.092735
3	0.010809	0.004867	0.107002	0.038087	0.33296	0.055261	0.589382	0.082265	0.713615	0.073886
6	0.003976	0.001121	0.172045	0.09656	0.335604	0.083948	0.71482	0.208258	0.865347	0.139821
24	0.013156	0.00746	0.011894	0.039228	0.079928	0.035965	0.301582	0.134414	0.545798	0.098682

Table 22: Strand scission factors (SSF) in animal AE28 coelomocytes over a 24-h period of recovery after acute exposure to UV-C or H_2O_2. Mean and standard error of n=2-4 technical replicates.

**AE28** *E. l. lucunter*	UV (J/m^2) 0		250		1000		3000		9999	
Recovery time (h)	mean	s.e.m.	mean	s.e.m.	mean	s.e.m.	mean	s.e.m.	mean	s.e.m.
0	0.066581	0.183713	0.410597	0.277529	0.349643	0.246661	0.170305	0.11131	0.093862	0.211824
1	0.002456	0.046228	-0.05902	0	-0.15829	0	0.637094	0.390461	0.394053	0.15919
3	0.005396	0.068603	-0.02352	0.108991	-0.10669	0.07981	0.061683	0.315189	-0.25507	0.058321
6	0.013846	0.080326	0.258474	0.32396	-0.02825	0.048191	0.327248	0.323587	0.604322	0.13457
24	0.001855	0.028462	0.184949	0.110834	0.442078	0.200976	0.752535	0.108947	1.051657	0.29614
	H_2O_2 (mM) 0		0.1		1		10		100	
Recovery time (h)	mean	s.e.m.	mean	s.e.m.	mean	s.e.m.	mean	s.e.m.	mean	s.e.m.
0	0.020409	0.091813	0.496602	0.134124	0.804476	0.105172	1.541822	0.308863	1.036677	0.067853
1	0.043146	0.114928	0.288508	0.051584	1.030167	0.194838	1.182451	0.083684	1.113707	0.121011
3	0.000157	0.008231	0.042824	0.038581	0.542434	0.057504	0.743195	0.037457	0.760522	0.048266
6	0.003214	0.036906	0.053285	0.006784	0.621981	0.052228	0.981057	0.007054	1.478675	0.246052
24	8.97E-05	0.006243	-0.04162	0.002926	0.170411	0.004948	-0.0574	0.038887	0.941665	0.047793

Table 23: Strand scission factors (SSF) in animal AE29 coelomocytes over a 24-h period of recovery after acute exposure to UV-C or H_2O_2. Mean and standard error of n=2-4 technical replicates.

**AE29** *E. l. lucunter*	UV (J/m^2) 0		250		1000		3000		9999	
Recovery time (h)	mean	s.e.m.	mean	s.e.m.	mean	s.e.m.	mean	s.e.m.	mean	s.e.m.
0	0.125582	0.346395	0.4139	0.173521	0.944626	0.419671	0.788453	0.570665	1.984041	0
1	0.009526	0.063409	0.191301	0.073433	0.57223	0.192208	0.463588	0.044331	0.783623	0.158031
3	0.002439	0.03291	0.342897	0.113579	0.792853	0.310636	0.438068	0.011574	1.563779	1.119493
6	0.030498	0.11963	0.163438	0.156474	0.151947	0.150904	0.43637	0.0758	0.869368	0.006777
24	0.002276	0.044501	0.471373	0.191029	0.05232	0.08446	0.746551	0.245259	0.855776	0.20776
	H_2O_2 (mM) 0		0.1		1		10		100	
Recovery time (h)	mean	s.e.m.	mean	s.e.m.	mean	s.e.m.	mean	s.e.m.	mean	s.e.m.
0	0.000761044	0.025718	0.283171	0.03159	0.68472	0.044233	0.870047	0.037423	0.857823	0.071266
1	0.004361612	0.044331	0.4822	0.093367	0.609353	0.009894	0.893028	0.025173	1.049764	0.024367
3	0.009127213	0.063706	0.273149	0.038543	0.514662	0.060609	0.923156	0.022528	1.121062	0.11225
6	0.000307926	0.016356	0.393194	0.052705	0.6043	0.053939	0.902374	0.034544	1.058578	0.012901
24	0.001690633	0.027394	-0.07288	0.040257	0.103631	0.058264	0.156285	0.105128	0.633207	0.053457

Table 24: Strand scission factors (SSF) in animal AE30 coelomocytes over a 24-h period of recovery after acute exposure to UV-C or H_2O_2. Mean and standard error of n=2-4 technical replicates.

**AE30** *E. l. lucunter*	UV (J/m^2) 0		250		1000		3000		9999	
Recovery time (h)	mean	s.e.m.	mean	s.e.m.	mean	s.e.m.	mean	s.e.m.	mean	s.e.m.
0	0.003098	0.036876	0.082911	0.101369	0.60352	0.079852	0.30756	0.12019	0.663525	0.247685
1	0.000677	0.017252	0.1525	0.091621	0.263684	0.072776	0.659973	0.284401	0.536154	0.136068
3	0.049613	0.155951	0.309591	0.140097	0.601183	0.359156	0.276728	0.004603	1.53871	0
6	0.084422	0.279644	-0.03614	0.141082	1.017679	0.308482				
24	0.016108	0.082563	0.021593	0.039791	0.3589	0.175503	0.313733	0.091546	0.364248	0.098105
	H_2O_2 (mM) 0		0.1		1		10		100	
Recovery time (h)	mean	s.e.m.	mean	s.e.m.	mean	s.e.m.	mean	s.e.m.	mean	s.e.m.
0	0.001406016	0.034965	-0.02859	0.041295	0.503508	0.118581	0.701801	0.171899	0.994796	0.105692
1	0.078156517	0.204	0.14146	0.129341	0.552237	0.133174	0.53772	0.110376	0.730243	0.159408
3	0	0	-0.0195	0.04292	0.288137	0.029176	0.373544	0.039	0.418707	0.056775
6	3.25057E-05	0.005314	0.040177	0	0.313551	0.033172	0.408143	0.119548	0.815043	0.041919
24	0.017174348	0.122944	-0.04746	0.144042	-0.0374	0	0.293974	0.055693	0.505044	0.086759

Table 25: Strand scission factors (SSF) in animal AE31 coelomocytes over a 24-h period of recovery after acute exposure to UV-C or H_2O_2. Mean and standard error of n=2-4 technical replicates.

**AE31** *E. l. lucunter*	UV (J/m^2) 0		250		1000		3000		9999	
Recovery time (h)	mean	s.e.m.	mean	s.e.m.	mean	s.e.m.	mean	s.e.m.	mean	s.e.m.
0	0.048663	0.209452	0.191007	0.137836	-0.18848	0.122338	0.072078	0.073228	0.365874	0.103185
1	0.039479	0.134817	0.120671	0.125856	0.456777	0.20177	0.267554	0.049093	1.432123	0.394239
3	0.011568	0.072749	0.14389	0.181452	-0.04238	0.084538	-0.0446	0.081187	0.096389	0.036249
6	0.000903	0.028018	0.258084	0.140635	0.089411	0.125883	0.394153	0.042653	0.815462	0.104846
24	0.004648	0.063652	0.280785	0.07772	0.246579	0.155461	-0.09329	0.027181	-0.16364	0.090549
	H_2O_2 (mM) 0		0.1		1		10		100	
Recovery time (h)	mean	s.e.m.	mean	s.e.m.	mean	s.e.m.	mean	s.e.m.	mean	s.e.m.
0	0.009090232	0.064607	0.072749	0.05977	0.696985	0.043271	0.883968	0.020562	0.834442	0.018058
1	0.00945262	0.064984	0.147288	0.087572	0.544613	0.037875	0.589954	0.078782	0.866302	0.071895
3	0.014208007	0.076681	0.067733	0.083982	0.371217	0.073575	0.475805	0.025137	0.723084	0.03438
6	0.007202718	0.079315	-0.20026	0.070383	-0.00179	0.094884	0.205517	0.054512	0.360619	0.081147
24	0.009049119	0.063764	0.113813	0.015714	-0.10654	0.121171	0.117482	0.007614	0.27468	0.032988

Table 26: Strand scission factors (SSF) in animal AE32 coelomocytes over a 24-h period of recovery after acute exposure to UV-C or H_2O_2. Mean and standard error of n=2-4 technical replicates.

**AE32** *E. l. lucunter*	UV (J/m^2) 0		250		1000		3000		9999	
Recovery time (h)	mean	s.e.m.	mean	s.e.m.	mean	s.e.m.	mean	s.e.m.	mean	s.e.m.
0	0.028388	0.117171	-0.07915	0	0.189998	0.001504	0.406958	0.151275	0.517161	0.106854
1	0.054632	0.153393	0.259361	0.390371	0.463454	0.52317	-0.05772	0.094619	0.258168	0.142274
3	0.008467	0.062056	0.232694	0.163674	0.185419	0.121729	0.288976	0.283461	0.706939	0.176418
6	0.005673	0.049509	0.113698	0.025709	-0.0254	0.12971	0.3289	0.067897	0.554131	0.152829
24	0.01851	0.093205	0.1641	0.012015	0.31867	0.066358	0.159723	0.150588	0.525417	0.041225
	H_2O_2 (mM) 0		0.1		1		10		100	
Recovery time (h)	mean	s.e.m.	mean	s.e.m.	mean	s.e.m.	mean	s.e.m.	mean	s.e.m.
0	0.010365	0.06805	0.319263	0.050141	0.725849	0.097076	0.917194	0.055986	1.0027	0.074315
1	0.002872	0.034884	-0.09882	0.045195	0.172779	0.035447	0.666534	0.081522	0.710429	0.147666
3	0.00196	0.029512	-0.00032	0.100566	0.059173	0.108877	0.291776	0.028634	0.501364	0.067628
6	0.007722	0.05931	0.133378	0.055593	0.257534	0.062521	0.607064	0.072661	0.854722	0.045387
24	0.006465	0.053689	0.072777	0.083929	0.169571	0.096517	0.313483	0.013114	0.615356	0.061086

Table 27: Strand scission factors (SSF) in animal AE33 coelomocytes over a 24-h period of recovery after acute exposure to UV-C or H_2O_2. Mean and standard error of n=2-4 technical replicates.

**AE33** *E. l. lucunter*	UV (J/m^2) 0		250		1000		3000		9999	
Recovery time (h)	mean	s.e.m.	mean	s.e.m.	mean	s.e.m.	mean	s.e.m.	mean	s.e.m.
0	0.003388	0.054318	0.047129	0.121708	-0.12915	0.064866	-0.04065	0.042602	0.13149	0.080524
1	0.019785	0.091655	-0.0811	0.054897	0.16574	0.091122	0.020222	0.049747	0.401656	0.207907
3	0.068546	0.250472	0.124128	0	-0.01326	0.007743	0.172039	0.03901	0.110288	0.0415
6	0.568284	0.86116	0.056631	0.304098	-0.01294	0.121587	-0.75565	0	0.032029	0.272477
24	0	0	0.16933	0.076554	0.066041	0.108598	-0.03702	0.026533	-0.08946	0.092255
	H_2O_2 (mM) 0		0.1		1		10		100	
Recovery time (h)	mean	s.e.m.	mean	s.e.m.	mean	s.e.m.	mean	s.e.m.	mean	s.e.m.
0	0.009557391	0.065217	0.005571	0.103306	0.408116	0.055407	0.851096	0.007746	0.645707	0.054606
1	0.036582721	0.132826	-0.02075	0.040876	0.07555	0.104114	0.346768	0.062281	0.384676	0.02654
3	0.01658542	0.12079	0.242484	0.062227	0.30952	0.044739	0.499022	0.154981	0.780601	0.046504
6	0.003545182	0.03915	0.173336	0	0.15381	0.036233	0.367469	0.049159	0.450342	0.099316
24	0.000285422	0.015747	0.064083	0.049442	0.163404	0.072301	0.227432	0.058911	0.111405	0.063241

Table 28: Strand scission factors (SSF) in animal AE34 coelomocytes over a 24-h period of recovery after acute exposure to UV-C or H_2O_2. Mean and standard error of n=2-4 technical replicates.

**AE34** *E. l. lucunter*	UV (J/m^2) 0		250		1000		3000		9999	
Recovery time (h)	mean	s.e.m.	mean	s.e.m.	mean	s.e.m.	mean	s.e.m.	mean	s.e.m.
0	0.003684	0.05665	0.037895	0.050528	0.102266	0.081315	0.20954	0.079875	0.41299	0.089216
1	0.018628	0.093762	0.333056	0.121033	0.404245	0.035253	0.900329	0.166037	0.762265	0.193828
3	0.026711	0.153883	0.264078	0.105592	0.704216	0.187237	1.126851	0.293339	1.104962	0.202121
6	0.001373	0.034552	0.267285	0.023456	1.101006	0.406025	1.08309	0.279238	1.320777	0.443523
24	0.02191	0.098429	0.693053	0.494249	0.186909	0.142864	0.569367	0.057406	0.123105	0.040994
	H_2O_2 (mM) 0		0.1		1		10		100	
Recovery time (h)	mean	s.e.m.	mean	s.e.m.	mean	s.e.m.	mean	s.e.m.	mean	s.e.m.
0	0.014563839	0.078618	0.067263	0.079601	0.312538	0.0512	0.732689	0.038001	0.681456	0.026268
1	0.002305666	0.031353	0.104905	0.021239	0.176675	0.01291	0.442952	0.025882	0.682777	0.050589
3	0.003377178	0.038915	0.097818	0.021002	0.214034	0.048229	0.529213	0.030912	0.684152	0.011697
6	0.00220316	0.030901	0.071317	0.079107	0.161685	0.024385	0.256564	0.03661	0.608517	0.030896
24	0.007007126	0.056029	0.170001	0.089844	0.090332	0.079532	0.163626	0.029054	0.42621	0.046601

Table 29: Strand scission factors (SSF) in animal AE35 coelomocytes over a 24-h period of recovery after acute exposure to UV-C or H_2O_2. Mean and standard error of n=2-4 technical replicates.

**AE35** *E. l. lucunter*	UV (J/m^2) 0		250		1000		3000		9999	
Recovery time (h)	mean	s.e.m.	mean	s.e.m.	mean	s.e.m.	mean	s.e.m.	mean	s.e.m.
0										
1										
3										
6										
24										
	H_2O_2 (mM) 0		0.1		1		10		100	
Recovery time (h)	mean	s.e.m.	mean	s.e.m.	mean	s.e.m.	mean	s.e.m.	mean	s.e.m.
0	0.05965	0.171858	0.193743	0.173341	0.264307	0.05886	1.631943	0	1.127063	0.29792
1	0.036354	0.133876	0.03539	0.061302	0.403782	0.195423	1.160516	0.07886	1.158087	0.271749
3	0.04106	0.139793	0.151823	0.08838	0.364501	0.048213	0.879343	0.006759	0.719427	0.042037
6	0.00758	0.056817	0.711937	0.125053	0.573764	0.014481	1.990373	0.304138	1.29628	0.409851
24	0.063484	0.173759	-0.16357	0.01833	0.086013	0.161617	1.197772	0	0.858821	0.166383

Table 30: Strand scission factors (SSF) in *Isostichopus badionotus* coelomocytes over a 24-h period of recovery after acute exposure to UV-C or H_2O_2. Combined data from n=8 individuals.

**AE15** *I. badionotus*	UV (J/m^2) 0		250		1000		3000		9999	
Recovery time (h)	mean	s.e.m.	mean	s.e.m.	mean	s.e.m.	mean	s.e.m.	mean	s.e.m.
0	0.003416	0.000586	0.011583	0.012596	0.022314	0.020993	0.033182	0.012813	0.035919	0.022274
1	0.003981	0.001188	0.019925	0.013629	0.04411	0.019502	0.061067	0.020363	0.040732	0.017127
3	0.001142	0.000292	0.018085	0.015353	0.032736	0.024133	0.051798	0.022836	0.032574	0.023772
6	0.003419	0.00131	0.047976	0.018507	0.073895	0.021943	0.113148	0.032909	0.087048	0.032819
24	0.001756	0.000709	-0.00762	0.02159	0.005178	0.02428	0.007866	0.02271	-0.00881	0.021008
	H_2O_2 (mM) 0		0.1		1		10		100	
Recovery time (h)	mean	s.e.m.	mean	s.e.m.	mean	s.e.m.	mean	s.e.m.	mean	s.e.m.
0	0.003981	0.001394	0.026914	0.022328	0.064838	0.009674	0.083915	0.012832	0.023773	0.026183
1	0.001342	0.00073	0.00946	0.024353	0.045551	0.01922	0.060518	0.032145	0.05872	0.024301
3	0.001981	0.001264	0.031242	0.017502	0.03536	0.007627	0.090234	0.015918	0.02942	0.01351
6	0.002965	0.000919	0.008555	0.017756	0.033878	0.016303	0.078444	0.013556	0.079042	0.034439
24	0.001701	0.000434	0.006554	0.006753	0.028654	0.011118	-0.00124	0.021119	-0.00791	0.034106

Table 31: Strand scission factors (SSF) in animal AE15 coelomocytes over a 24-h period of recovery after acute exposure to UV-C or H_2O_2. Mean and standard error of n=2-4 technical replicates.

**AE15** *I. badionotus*	UV (J/m^2) 0		250		1000		3000		9999	
Recovery time (h)	mean	s.e.m.	mean	s.e.m.	mean	s.e.m.	mean	s.e.m.	mean	s.e.m.
0	0.012984	0.060611	0.08325	0.036159	-0.00032	0.025552	0.183643	0.047341	0.076974	0.026602
1	0.010639	0.0667	0.042561	0.045338	0.097816	0.06492	0.089509	0.027198	0.108494	0.041865
3	0.002869	0.032869	0.023625	0.056853	0.018675	0.071683	0.06687	0.024658	-0.00141	0.043127
6	0.006939	0.053922	0.088969	0.047165	0.187103	0.136619	0.372435	0.047072	0.385259	0.013606
24	0.007417	0.056414	-0.08641	0.036552	-0.16997	0.036707	-0.14774	0.012363	-0.07283	0.022275
	H_2O_2 (mM) 0		0.1		1		10		100	
Recovery time (h)	mean	s.e.m.	mean	s.e.m.	mean	s.e.m.	mean	s.e.m.	mean	s.e.m.
0	0.000662	0.013867	0.097443	0.0528	0.104671	0.055807	0.141579	0.033286	0.361175	0.02934
1	0.00255	0.027464	-0.11735	0.018489	-0.00505	0.023554	-0.04928	0.043785	0.308833	0.027897
3	0.004854	0.037521	-0.008	0.033795	0.036737	0.02956	0.015372	0.037351	0.101981	0.020044
6	0.000501	0.014843	0.063634	0.064516	0.126673	0.017461	0.205425	0.00595	0.43931	0.011108
24	0.002727	0.034384	-0.02632	0.043444	0.018827	0.017944	-0.0059	0.060473	0.118896	0.022728

Table 32: Strand scission factors (SSF) in animal AE16 coelomocytes over a 24-h period of recovery after acute exposure to UV-C or H_2O_2. Mean and standard error of n=2-4 technical replicates.

**AE16** *I. badionotus*	UV (J/m^2) 0		250		1000		3000		9999	
Recovery time (h)	mean	s.e.m.	mean	s.e.m.	mean	s.e.m.	mean	s.e.m.	mean	s.e.m.
0	0.008037	0.047926	0.100966	0.020155	0.108345	0.038116	0.177278	0.035688	0.237323	0.005353
1	0.035633	0.029585	0.052201	0.007696	0.072152	0.014668	0.031293	0.176396	0.177601	0.009196
3	0.024011	0.137294	0.249379	0.038031	0.189784	0.146746	0.333336	0.032701	0.376683	0.039001
6	0.019503	0.13113	0.088079	0.047404	0.161744	0.037939	0.237214	0.016524	0.164189	0.043731
24	0.001391	0.024608	-0.12771	0.030852	-0.01539	0.028503	0.008941	0.021887	0.024028	0.022311
	H_2O_2 (mM) 0		0.1		1		10		100	
Recovery time (h)	mean	s.e.m.	mean	s.e.m.	mean	s.e.m.	mean	s.e.m.	mean	s.e.m.
0	0.026664	0.086195	0.310365	0.073171	0.194111	0.022375	0.368075	0.053537	0.368687	0.053751
1	0.116168	0.063612	0.111223	0.051027	0.075727	0.016506	0.006186	0.170263	0.210215	0.022101
3	-4.1E-08		0.045548	0.0169	-0.00596	0.026164	0.145504	0.015861	0.03565	0.031246
6	0.005792	0.07109	0.087713	0.022112	0.084654	0.034287	0.116917	0.010385	0.119446	0.019365
24	0.006819	0.053619	-0.02229	0.013045	-0.00101	0.018286	0.047018	0.023778	-0.03487	0.01666

Table 33: Strand scission factors (SSF) in animal AE17 coelomocytes over a 24-h period of recovery after acute exposure to UV-C or H_2O_2. Mean and standard error of n=2-4 technical replicates.

**AE17** *I. badionotus*	UV (J/m^2) 0		250		1000		3000		9999	
Recovery time (h)	mean	s.e.m.	mean	s.e.m.	mean	s.e.m.	mean	s.e.m.	mean	s.e.m.
0	0.004622	0.044968	0.008853	0.023663	-0.02008	0.018014	0.014342	0.066149	-0.06632	0.025283
1	0.001807	0.028294	-0.09267	0.023592	0.021063	0.029676	-0.00328	0.024452	-0.04073	0.039121
3	0.020517	0.097423	-0.08382	0.002789	-0.08734	0.042517	-0.10471	0.012008	-0.11064	0.039426
6	0.000802	0.018576	0.023111	0.025153	0.11229	0.007701	0.11434	0.035741	0.035815	0.01712
24	-1.7E-09	#DIV/0!	0.086836	0.038489	-0.04399	0.11014	0.027196	0.010035	-0.02162	0.012719
	H_2O_2 (mM) 0		0.1		1		10		100	
Recovery time (h)	mean	s.e.m.	mean	s.e.m.	mean	s.e.m.	mean	s.e.m.	mean	s.e.m.
0	0.001273	0.023518	0.164065	0.062621	0.16753	0.02577	0.217087	0.021313	0.072136	0.007191
1	0.001784	0.028068	-0.09942	0.087952	0.172784	0.043288	0.100938	0.052923	-0.00033	0.01243
3	0.027753	0.10663	0.244969	0.00093	0.077047	0.184082	0.228743	0.041647	0.012283	0.011804
6	0.001111	0.02201	0.014359	0.033377	0.09445	0.033668	0.155499	0.048829	0.022147	0.021782
24	0.006114	0.05132	0.048133	0.021276	0.071983	0.085868	-0.0916	0.035251	-0.18347	0.040212

Table 34: Strand scission factors (SSF) in animal AE18 coelomocytes over a 24-h period of recovery after acute exposure to UV-C or H_2O_2. Mean and standard error of n=2-4 technical replicates.

**AE18** *I. badionotus*	UV (J/m^2) 0		250		1000		3000		9999	
Recovery time (h)	mean	s.e.m.	mean	s.e.m.	mean	s.e.m.	mean	s.e.m.	mean	s.e.m.
0	0.00186	0.023512	-0.01019	0.016695	0.005936	0.040025	0.044465	0.038348	0.042678	0.020877
1	0.001451	0.020432	0.020833	0.068099	0.035481	0.024272	-0.03679	0.017597	-0.01429	0.034618
3	0.00363	0.032248	0.011117	0.025228	0.019753	0.044656	-0.04364	0.007678	-0.07305	0.006487
6	0.003606	0.039085	0.07215	0.021324	-0.00862	0.021951	-0.02365	0.014282	-0.00442	0.032876
24	0.003734	0.033439	-0.01441	0.028869	-0.01016	0.04415	-0.09257	0.024022	-0.10196	0.000976
	H_2O_2 (mM) 0		0.1		1		10		100	
Recovery time (h)	mean	s.e.m.	mean	s.e.m.	mean	s.e.m.	mean	s.e.m.	mean	s.e.m.
0	0.001698	0.022076	0.02326	0.042283	0.034082	0.086876	0.013427	0.039442	-0.05877	0.053085
1	0.002904	0.029381	-0.04891	0.018179	-0.01904	0.013372	0.045727	0.026474	0.020076	0.085025
3	2.69E-05	0.003412	0.062194	0.028316	0.073719	0.048149	0.097343	0.068709	-0.00106	0.048742
6	0.011817	0.073576	0.006559	0.031536	0.02979	0.04327	0.060968	0.020327	-0.01403	0.026457
24	0.003705	0.039616	0.022525	0.011838	-0.02042	0.031658	-0.10837	0.029134	-0.16313	0.006547

Table 35: Strand scission factors (SSF) in animal AE19 coelomocytes over a 24-h period of recovery after acute exposure to UV-C or H_2O_2. Mean and standard error of n=2-4 technical replicates.

**AE19** *I. badionotus*	UV (J/m^2) 0		250		1000		3000		9999	
Recovery time (h)	mean	s.e.m.	mean	s.e.m.	mean	s.e.m.	mean	s.e.m.	mean	s.e.m.
0	0.013269	0.059808	-0.05127	0.035042	-0.08096	0.056206	-0.01865	0.026426	-0.05058	0.033294
1	0.021918	0.094839	0.024984	0.004887	-0.065	0.140675	0.062564	0.058253	-0.0004	0.026648
3	0.001905	0.023481	0.006146	0.040026	0.046538	0.032173	0.134765	0.018642	0.029583	0.020662
6	0.003314	0.038363	-0.06822	0.059479	-0.05292	0.021481	-0.01958	0.048606	-0.06915	0.005797
24	0.000531	0.015271	-0.04873	0.047234	-0.04588	0.013135	-0.03046	0.083467	-0.14957	0.033741
	H_2O_2 (mM) 0		0.1		1		10		100	
Recovery time (h)	mean	s.e.m.	mean	s.e.m.	mean	s.e.m.	mean	s.e.m.	mean	s.e.m.
0	0.019642	0.074022	-0.13467	0.109153	0.10631	0.026469	0.144412	0.03795	-0.04512	0.018283
1	0.004752	0.037213	-0.01154	0.03571	-0.00872	0.01857	-0.04161	0.016723	-0.05904	0.046755
3	0.000474	0.011685	-0.03745	0.043756	0.043152	0.022809	0.062883	0.029308	-0.00067	0.007723
6	0.002456	0.046227	-0.11638	0.112523	-0.00669	0.050722	0.056007	0.039995	-0.01265	0.039657
24	0.005791	0.051121	-0.01813	0.019578	0.05275	0.051741	-0.03917	0.026573	-0.12189	0.011589

Table 36: Strand scission factors (SSF) in animal AE20 coelomocytes over a 24-h period of recovery after acute exposure to UV-C or H_2O_2. Mean and standard error of n=2-4 technical replicates.

**AE20** *I. badionotus*	UV (J/m^2) 0		250		1000		3000		9999	
Recovery time (h)	mean	s.e.m.	mean	s.e.m.	mean	s.e.m.	mean	s.e.m.	mean	s.e.m.
0	-0.14059	0.075671	-0.16068	0.025159	-0.12452	0.009783	-0.11334	0.005394	-0.12881	0.039283
1	0.003802	0.032909	0.009337	0.030243	0.043886	0.043963	0.084349	0.013293	0.072594	0.013768
3	0.003783	0.032375	-0.00723	0.032117	0.019456	0.008647	0.044819	0.004232	0.047001	0.021489
6	0.002225	0.031014	0.166666	0.024508	0.158925	0.015101	0.214912	0.030846	0.134833	0.024224
24	0.002126	0.030063	0.040124	0.002761	0.03682	0.015123	0.085387	0.022123	0.0419	0.008794
	H_2O_2 (mM) 0		0.1		1		10		100	
Recovery time (h)	mean	s.e.m.	mean	s.e.m.	mean	s.e.m.	mean	s.e.m.	mean	s.e.m.
0	0.012684	0.05946	0.030119	0.046313	0.059462	0.035191	0.107523	0.022792	-0.01726	0.026532
1	0.00738	0.057973	0.079376	0.043609	0.072681	0.02818	0.083334	0.030332	0.093227	0.032903
3	0.002788	0.03441	0.014668	0.022735	0.042141	0.051323	0.126678	0.021354	0.033424	0.036097
6	0.001088	0.021596	0.029951	0.017231	0.089329	0.019788	0.107291	0.029874	0.130717	0.023951
24	0.003931	0.041039	0.036186	0.04534	0.061194	0.021759	-0.01448	0.034193	-0.01297	0.011922

Table 37: Strand scission factors (SSF) in animal AE21 coelomocytes over a 24-h period of recovery after acute exposure to UV-C or H_2O_2. Mean and standard error of n=2-4 technical replicates.

**AE21** *I. badionotus*	UV (J/m^2) 0		250		1000		3000		9999	
Recovery time (h)	mean	s.e.m.	mean	s.e.m.	mean	s.e.m.	mean	s.e.m.	mean	s.e.m.
0	0.00256	0.027461	0.092939	0.016318	0.077105	0.018808	0.060779	0.010865	0.093475	0.025425
1	0.012496	0.042202	0.02298	0.015416	0.08988	0.040553	0.127362	0.015559	0.112243	0.039941
3	0.000435	0.01944	0.149459	0.008322	0.217555	0.02649	0.226119	0.012233	0.207275	0.014702
6	0.003612	0.040198	0.04119	0.032688	0.105155	0.038786	0.166065	0.007748	0.154087	0.025898
24	0.002313	0.032018	0.057832	0.005928	0.061317	0.039703	0.040211	0.009506	0.040028	0.007297
	H_2O_2 (mM) 0		0.1		1		10		100	
Recovery time (h)	mean	s.e.m.	mean	s.e.m.	mean	s.e.m.	mean	s.e.m.	mean	s.e.m.
0	0.002567	0.027378	0.002484	0.02068	0.129189	0.046264	0.121697	0.0292	0.022838	0.014573
1	0.001757	0.027487	0.170584	0.051621	0.179595	0.03306	0.342275	0.025756	0.19613	0.014412
3	0.00155	0.026063	0.036212	0.061128	0.07931	0.054788	0.201994	0.014039	0.14356	0.008067
6	0.006123	0.051493	0.018436	0.037904	0.048626	0.047436	0.100128	0.009368	0.157712	0.008252
24	0.000207	0.013409	0.027525	0.037154	0.111391		0.024105	0.053907	0.104517	0.040448

Table 38: Strand scission factors (SSF) in animal AE22 coelomocytes over a 24-h period of recovery after acute exposure to UV-C or H_2O_2. Mean and standard error of n=2-4 technical replicates.

**AE22** *I. badionotus*	UV (J/m^2) 0		250		1000		3000		9999	
Recovery time (h)	mean	s.e.m.	mean	s.e.m.	mean	s.e.m.	mean	s.e.m.	mean	s.e.m.
0	0.003957	0.041049	0.024259	0.078117	0.179737	0.013061	0.045709	0.096874	0.183005	0.008637
1	0.00391	0.034006	0.056468	0.015254	0.164065	0.040604	0.197989	0.054831	0.073622	0.012445
3	0.001843	0.028194	0.008776	0.080752	0.090948	0.030144	0.064177	0.015811	0.01695	0.017047
6	0.005975	0.050353	0.077125	0.032103	0.126207	0.012971	0.176213	0.016492	0.142279	0.019598
24	0.009901	0.064959	0.048821	0.025129	0.180469	0.032618	0.138781	0.024314	0.093069	0.009531
	H_2O_2 (mM) 0		0.1		1		10		100	
Recovery time (h)	mean	s.e.m.	mean	s.e.m.	mean	s.e.m.	mean	s.e.m.	mean	s.e.m.
0	0.001418	0.020543	0.064759	0.016071	0.139687	0.049041	0.145159	0.05412	0.010834	0.014559
1	0.009031	0.018554	0.055643	0.010015	0.053337	0.028201	0.123519	0.020252	0.07521	0.008246
3	0.000661	0.013917	0.054134	0.016725	0.068716	0.027447	0.134733	0.010557	0.015986	0.007432
6	0.011839	0.072893	0.013096	0.044002	-0.04471	0.044335	0.124611	0.007196	0.084863	0.022671
24	0.000312	0.011664	0.007046	0.011879	0.042872	0.033291	0.149817	0.033225	0.186761	0.025671
